# Effects of physical activity on health-related outcomes in Sjögren’s syndrome: a systematic review and meta-analysis of randomized controlled trials

**DOI:** 10.3389/fimmu.2026.1753791

**Published:** 2026-04-07

**Authors:** Qianfei Gao, Congxian Fan, Yeyun Zhang, Zehui Lian, Haojie Li, Meng Zhang

**Affiliations:** 1 School of Sports and Health, Anhui University of Applied Technology, Hefei, China; 2 School of Physical Education, Huangshan University, Huangshan, China; 3 School of Physical Education, Guangxi Minzu Normal University, Chongzuo, China; 4 School of Exercise and Health, Shanghai University of Sport, Shanghai, China

**Keywords:** Sjögren's syndrome, physical activity, meta-analysis, cardiopulmonary function, pain

## Abstract

**Introduction:**

Individuals with Sjögren's syndrome (SjD) experience notable health challenges, including chronic pain, fatigue, and depression; however, the potential benefits of physical activity in alleviating these issues remain unclear. We aim to systematically assess the comprehensive effects of physical activity on individuals with SjD.

**Methods:**

A comprehensive literature search was conducted in Medline, Embase, Scopus, Web of Science, Cochrane Library, SPORTDiscus, and ClinicalTrials.gov for studies published from inception to November 2025. Two independent reviewers screened the search results and extracted the data. Effect sizes were calculated as the standardized mean difference (SMDs) with 95% confidence intervals (CIs) using random-effects models. Methodological quality was assessed using the Cochrane Collaboration Risk of Bias Tool 2.0, and the certainty of evidence was evaluated with the GRADEpro online tool. Sensitivity, subgroup, and regression analyses were performed to explore potential sources of heterogeneity.

**Results:**

This analysis included six studies with a total of 277 participants with SjD. Compared to controls, physical activity interventions significantly improved cardiopulmonary function (SMD 0.59 [95% CI 0.20 to 0.99]), functional capacity (SMD 0.69 [95% CI 0.33 to 1.05]), general health status (SMD 0.46 [95% CI 0.15 to 0.76]), vitality (SMD 0.51 [95% CI 0.15 to 0.86]), and mental health (SMD 0.42 [95% CI 0.13 to 0.72]). However, no significant improvements were observed in pain, social aspects, fatigue, and the EULAR Sjögren's Syndrome Disease Activity Index.

**Discussion:**

The results highlight aerobic and resistance training are regarded as effective and practical exercise options.

**Systematic Review Registration:**

https://www.crd.york.ac.uk/PROSPERO/, identifier CRD42024513141.

## 1 Introduction

Sjögren’s syndrome (SjD) is a prototypical autoimmune disorder that predominantly affects middle-aged women, with peak incidence occurring between the ages of 40 and 60 (1, 2). It is characterized by a broad spectrum of systemic manifestations, with hallmark symptoms including severe xerosis (dryness of the skin, eyes, and mucosal surfaces), chronic widespread pain, sleep disturbances, and a heightened prevalence of anxiety and depression (3). The estimated prevalence of SjD is approximately 0.5%, with a striking female-to-male ratio of 9:1. Despite being traditionally considered a non-life-threatening condition, SjD carries a notable disease burden, with an annual mortality rate of approximately 4 per 1,000 individuals (4, 5).

The pathogenesis of SjD remains incompletely understood, though a strong familial genetic predisposition has been identified (1). Dysregulated immune pathways, including aberrant B- and T-cell activation, type I interferon signaling, and increased pro-inflammatory mediators—such as B-cell activating factor, interleukin-6, interleukin-17, and tumor necrosis factor—are implicated in the pathogenesis of SjD (6–8). In addition to genetic susceptibility, various environmental factors, including infections, pharmacological agents, and other immune system disturbances, contribute to the disruption of both innate and adaptive immune responses (9). Given the multifaceted nature of SjD, current therapeutic strategies primarily focus on symptom management and inflammation control. Clinical guidelines from the European League Against Rheumatism and the American College of Rheumatology (ACR) recommend pharmacological interventions such as muscarinic receptor agonists, glucocorticoids, antimalarials, immunosuppressants, intravenous immunoglobulins, and biologic agents (10, 11). Furthermore, the latest ACR guidelines highlight mycophenolate mofetil, azathioprine, rituximab, and cyclophosphamide as first-line treatments (11, 12). However, despite their widespread use, these therapies often yield inconsistent efficacy and are associated with adverse effects, including localized hypersensitivity, gastrointestinal complications, and dermatological reactions (13), underscoring the need for safer and more effective treatment approaches. In this context, physical activity has emerged as a promising, non-pharmacological intervention due to its well-documented health benefits, safety profile, and cost-effectiveness (14). Physical activity research has demonstrated that regular physical activity reduces the risk of cardiovascular diseases (15), stroke (16), diabetes (17), hypertension (18), obesity (19), and certain cancers (20), with minimal adverse effects when performed at appropriate intensities. Moreover, physical activity programs can be tailored to accommodate diverse age groups and functional capacities, making them an accessible and adaptable therapeutic option. Despite these advantages, clinical evidence supporting the efficacy of physical activity in SjD remains limited and fragmented, hindering its integration into standard care. Consequently, further investigation is warranted to establish physical activity as a viable adjunctive therapy for improving disease management and enhancing the quality of life in individuals with SjD.

Despite the growing recognition of physical activity as a potential adjunctive therapy for SjD, research on its effectiveness remains limited. Existing randomized controlled trials (RCTs) evaluating physical activity interventions for SjD often employ heterogeneous outcome measures, resulting in fragmented and inconsistent findings. Additionally, comprehensive data integration is lacking, making it difficult to draw definitive conclusions about the overall impact of physical activity on SjD management. To address these gaps, this meta-analysis aims to systematically evaluate the effects of physical activity interventions on individuals with SjD. Cardiopulmonary function, functional capacity and pain were predefined as the primary outcomes, as these domains are recognized by the Outcome Measures in Rheumatology (21) group and the European Alliance of Associations for Rheumatology (EULAR) (10) as core outcomes of disease-related impairment in systemic autoimmune diseases. Secondary outcomes included general health status, vitality, social aspects, fatigue, mental health, and the EULAR Sjögren’s Syndrome Disease Activity Index (ESSDAI), reflecting the multidimensional symptom burden emphasized in international SjD guidelines (10). Furthermore, it seeks to explore the moderating influence of key covariates—such as region, sessions, sessions duration, length, and survey methods—through regression and subgroup analyses. By synthesizing existing evidence, this review aspires to establish a structured framework that can guide clinicians in optimizing physical activity interventions and inform future therapeutic strategies for improving patient outcomes in SjD care.

## 2 Methods

### 2.1 Protocol and registration

This study followed the Preferred Reporting Items for Systematic Review and Meta-analyses Statement (22). The protocol of this study was registered with the International Prospective Register of Systematic Reviews (PROSPERO registration number: CRD42024513141). In addition, ethical approval and patient consent were not required for this study, as all data used were based on previously published findings.

### 2.2 Search strategy

We systematically searched and identified relevant studies (from library inception to November 2025) using MEDLINE (via PubMed), Cochrane Library, Embase, Web of Science, Scopus, ClinicalTrials.gov, and SPORTDiscus, with no publication date or language limitations. The search terms including “cardiopulmonary function”, “physical activity”, “Sjögren’s syndrome”, and “Randomized Controlled Trials”. These keywords were used as MeSH headings and accessible text terms. The detailed search strategy is presented in **Appendix 1**.

### 2.3 Inclusion and exclusion criteria

Studies that met all of the following criteria were included:

1. The study population was patients with primary SjD, diagnosed according to established international criteria, including the 2002 American European Consensus Group criteria or the 2016 ACR/EULAR classification criteria, with no restriction on age.
2. Trials in which the physical activity intervention was compared with no treatment or any other treatment or procedure.
3. Control groups did not involve any structured or supervised physical activity interventions.
4. The primary efficacy outcomes included cardiopulmonary function, functional capacity, general health status, vitality, mental health, pain, social aspects, ESSDAI, and fatigue.
5. The type of study was a RCT.

Studies were excluded if the following conditions existed:

1. Patients with secondary SjD or other systemic autoimmune diseases (e.g., systemic lupus erythematosus, rheumatoid arthritis).
2. Intervention groups use multimodal interventions such as physical activity combined with psychotherapy or behavioral interventions.
3. Academic conferences, experimental programs, and systematic reviews.
4. Incorrect, missing, or unextractable information about the study data.

### 2.4 Study selection and data extraction

The retrieved studies were imported into Endnote X9 (Clarivate, Philadelphia, PA, USA), and duplicate trials were removed. Two independent authors reviewed the titles and abstracts of all potential citations according to the inclusion criteria, independently assessed study eligibility, and independently extracted relevant data from studies that met the inclusion criteria. Data extracted included study characteristics, participant characteristics, intervention characteristics, and measurements and outcomes. In case of any disagreement, it was discussed with the third independent author to reach a consensus. Data on study characteristics, participants, interventions, and outcomes were extracted using a standardized approach, allowing readers to appreciate potential differences in intervention effects across studies. When outcome data were missing or not directly reported, we first attempted to extract them from figures or **Supplementary Materials**. If unavailable, we contacted the corresponding author up to three times within 6 weeks; in the absence of a response, analyses were based on available data without imputation.

### 2.5 Risk of bias and credibility assessment

We assessed the risk of bias for the included studies using the Cochrane Risk of Bias 2 tool (23). To evaluate the credibility of the outcomes, we employed the Grading of Recommendations, Assessment, Development, and Evaluation (GRADE) approach (24). The overall quality of evidence for each outcome was summarized in a summary of findings table, which was generated using GRADEpro (Evidence Prime Inc., McMaster University, 2020). Two researchers independently summarized the findings, and any disagreements were resolved through discussion or by consulting a third researcher.

### 2.6 Summary measures and synthesis

All data were statistically analyzed and presented in R software (version 4.3.2) (25). For studies that met the inclusion criteria, the mean and standard deviation (SD) of the baseline and endpoints of the physical activity and control groups were extracted as the endpoints were all continuous variables. When standard errors were reported in the studies, we converted these data into SDs (26). When such data were unavailable, the means and SDs were derived from sample size, median, interquartile range, and minimum and maximum values (27). The mean and SD of the baseline and endpoint differences for the physical activity and control groups were used to calculate the relevant outcomes for each study. If a lower value represented a better study result, the result was multiplied by −1, as recommended in the Cochrane Handbook for Systematic Reviews of Interventions (28). The weight of each study was determined by the precision of its effect estimate, which was equal to the inverse of the variance. We derived standard mean differences (SMDs) with 95% confidence intervals (CIs) for the combined effect sizes of physical activity on cardiopulmonary function, functional capacity, general health status, vitality, pain, social aspects, fatigue, ESSDAI and mental health, using random effects model (29). The SMD effect sizes were quantified into four dimensions, i.e., large effect (≥0.8), moderate effect (0.5), small effect (0.2) (30). The percentage of variability in the combined estimates attributable to heterogeneity other than chance was assessed using the I^2^ statistic, and the degree of heterogeneity between studies could be classified into three dimensions, Low (0 percent to 25 percent), moderate (26 percent to 75 percent), and high (76 percent to 100 percent) (31). Statistical heterogeneity of trials was assessed using the Cochran Q statistic to test the original hypothesis that effect sizes did not differ between studies (32). The likelihood of publication bias can be judged by assessing the funnel plot (33) and Egger’s regression test (34).

### 2.7 Additional analyses

We performed sensitivity and meta-regression analyses to explore sources of heterogeneity and potential moderating effects of moderating variables on physical activity efficacy. Relevant covariates, both continuous and categorical, included region, sessions (sessions per week), length (intervention duration in weeks), session duration (minutes per session), and survey methods (measures used for outcome assessment). We used a randomized meta-regression model and restricted maximum likelihood to calculate the size effect of the effect estimates (35). Missing data were supplemented by average substitution (36).

## 3 Results

### 3.1 Study selection

**Figure 1:**
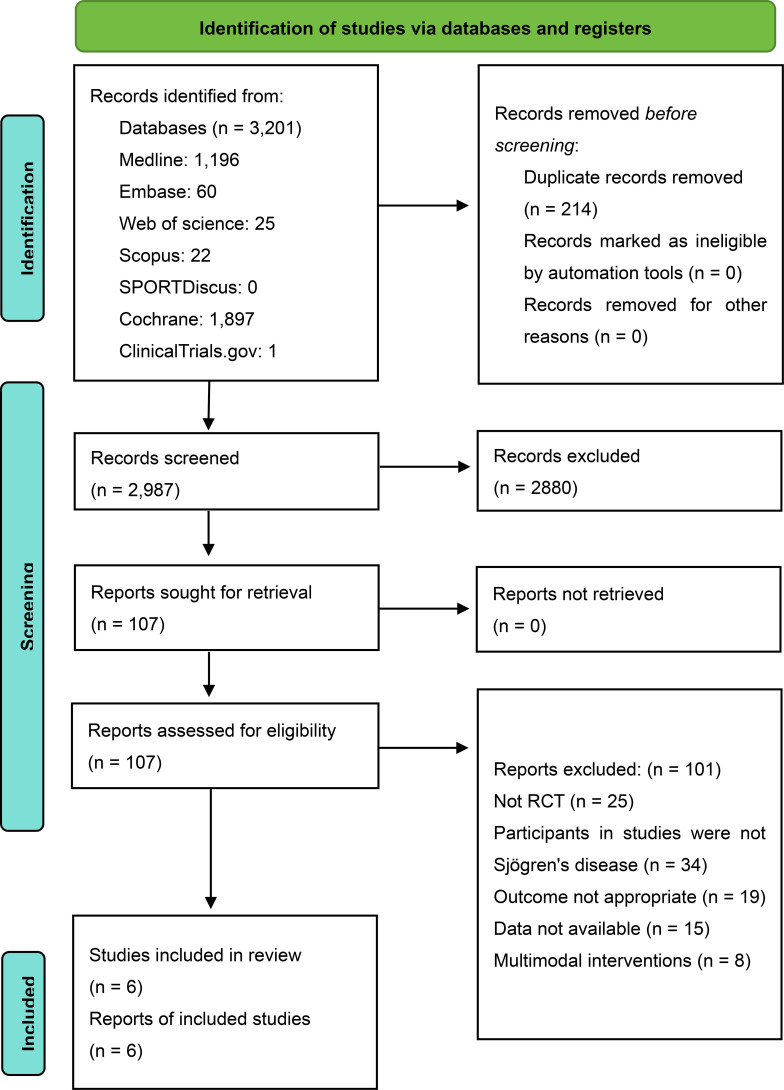
PRISMA flow diagram of study selection. RCT, Randomized controlled trial.

A total of 3,201 records were identified through database searches. After removing 214 duplicates, 2,987 articles underwent title/abstract screening. Finally, six studies were included in the meta-analysis (**Figure 1**).

### 3.2 Characteristics of included studies

The six studies involved 277 individuals (intervention group: n=141; control group: n=136). With most from South America (4/6, 66.67%), followed by Europe (1/6, 16.67%) and Asia (1/6, 16.67%). All individuals were female (age range: 46.08-62.7 years). Four studies used supervised physical activity interventions, and one reported adverse event. Aerobic and resistance training were the primary modalities. Outcomes included cardiopulmonary function (37–39), functional capacity (39–41), pain (38–41), general health status (39–42), vitality (39–41), social aspects (39–41), ESSDAI (38, 40, 41), fatigue (38, 39, 41), and mental health (38–41). The specific characteristics of the included studies are listed in **Table 1**.

**Table 1:** Characteristics of included studies.

Study	Sample size, Age (mean±SD)	Supervision	Disease duration (years)	Adverse	Length (weeks)	Sessions	Sessions duration	Treatment	Outcome
Garcia,2021, Brazil (37)	EG: 30, 60.43±12.2; CG: 30, 55.77±10.42	Yes	EG: 6.15±4.20; CG: 5.73±4.55	NR	28	2	45	EG: Resistance training; CG: Standard treatment protocol	1、
Minali,2020, Brazil (40)	EG: 26, 61.9±11.4; CG: 25, 56.6±10.3	Yes	NR	Joint pain	16	2	NR	EG: Resistance exercise; CG: Not exercises	1、3、4、5、6、7、9、
Miyamoto,2019, Brazil (38)	EG: 23, 53.4±8.6; CG: 22, 51.3±8.7	Yes	EG: 2±50.75; CG: 3.4±20.5	NR	16	3	35	EG: Walking exercise; CG: Not to regular physical exercise	1、3、7、8、
Dardin,2022, Brazil (41)	EG: 30, 62.7±12.95; CG: 26, 58.12±10.21	NR	EG: 6.15±4.20; CG: 5.73±4.55	NR	16	2	NR	EG: Resistance training; CG: Regular physical exercise	2、3、4、5、6、7、8、9、
Strömbeck,2007, Sweden (39)	EG: 9, 60±6; CG: 10, 56.5±5.25	Yes	EG: 5±3; CG: 8±5	NR	12	3	45	EG: Aerobic capacity; CG: Low-intensity home exercises	1、3、4、5、6、8、9、
Kurt,2023, Turkey (42)	EG: 23, 46.08±9.66; CG: 23, 49.47±8.55	NR	NR	NR	8	5	30	EG: Pelvic floor, diaphragm exercises; CG: Relaxation exercises	3、4、

CG, control group; EG, exercise group; NR, not report; Outcome, 1, Cardiopulmonary function; 2, Functional capacity; 3, Pain; 4, General health status; 5, Vitality; 6, Social aspects; 7, ESSDAI; 8, Fatigue; 9, Mental Health.

### 3.3 Quality assessment and evidence certainty

All studies implemented blinding, except one (39) that did not mention allocation concealment, leading to some concerns about bias. Missing outcome data, measurement, and reporting were rated as low risk (37–42) (**Supplementary Figures S1, S2** in **Appendix 2**). According to GRADE assessment, functional capacity, vitality, social aspects, and ESSDAI had moderate certainty; cardiopulmonary function, general health status, fatigue, and mental health showed low certainty; pain showed very low certainty. Downgrading was due to heterogeneity, small sample sizes and total population size is less than 200. (**Table 5** in **Appendix 3**).

### 3.4 Primary results

#### 3.4.1 Cardiopulmonary function

Three studies (37–39), encompassing 124 individuals, reported outcomes on cardiopulmonary function. Specific indicators extracted from these studies included VO_2_max and HRmax, which were combined to represent overall cardiopulmonary function for the purpose of meta-analysis (SMD 0.59 [95% CI 0.20 to 0.99]; Q = 8.22, df = 4, P = 0.084, I^2^ = 51%) (**Table 2**). Funnel plot symmetry indicated no significant publication bias (*Pegger* = 0.62) (**Supplementary Figure S5** in **Appendix 4**). Univariate regression analyses revealed no significant associations between the outcomes and any potential covariates (**Table 3**). Subgroup analyses stratified by region, sessions, length, sessions duration, and survey methods demonstrated that these covariates collectively influenced the efficacy of physical activity interventions. Detailed findings are presented in **Table 4**.

**Table 2:** Primary outcomes of included studies.

Meta-Analyses Variables	Outcomes	Number of Studies	EG	CG	SMD, 95% CI	I^2^(%)	P_heterogeneity_	Z-Value
Primary								
	Cardiopulmonary function	5	115	114	**0.59 (0.20 to 0.99)**	51	0.08	2.94
	Functional capacity	3	65	61	**0.69 (0.33 to 1.05)**	0	0.39	3.72
	Pain	5	111	106	0.32 (-0.09 to 0.72)	53	0.07	1.53
Secondary								
	General health status	4	88	84	**0.46 (0.15 to 0.76)**	0	0.68	2.93
	Vitality	3	65	61	**0.51 (0.15 to 0.86)**	0	0.69	2.79
	Social aspects	3	65	61	0.27 (-0.08 to 0.62)	0	0.81	1.52
	ESSDAI	3	65	61	-0.09 (-0.41 to 0.23)	0	0.88	-0.56
	Fatigue	4	71	68	-0.57 (-1.57 to 0.44)	85	<0.01	-1.11
	Mental health	8	162	154	**0.42 (0.13 to 0.72)**	45	0.08	2.80
Influence analysis								
	Cardiopulmonary function	4	85	84	**0.59 (0.09 to 0.71)**	0	0.01	2.55
	Pain	4	85	81	0.16 (-0.20 to 0.52)	15.6	0.37	0.89

CG, control group; CI, confidence interval; EG, exercise group; ESSDAI, EULAR Sjögren's syndrome disease activity index; SMD, standardised mean difference.

Bold values indicate that the 95% confidence interval (CI) does not cross zero, representing statistical significance.

**Table 3:** Primary results based on regression.

Regression analysis based on outcomes	Covariates	Number of studies	EG	CG	Coefficient, 95% CI	I^2^(%)	P	Z-Value	Adjusted R^2^(%)
Cardiopulmonary function	Region	5	115	114	0.61 (-0.69 to 0.82)	56.53	0.86	0.17	0.00
	Sessions	5	115	114	0.34 (-0.85 to 0.40)	50.84	0.40	-0.84	0.00
	Length	5	115	114	-0.15 (-0.61 to 0.31)	54.70	0.53	-0.63	0.00
	Sessions duration	5	115	114	0.06 (-0.01 to 0.13)	31.89	0.09	0.71	53.89
	Survey methods	5	115	114	-0.53 (-1.23 to 0.18)	38.17	0.14	-1.47	38.52
Functional capacity	Region	3	65	61	0.76 (-0.33 to 1.85)	0.00	0.17	1.36	100.00
	Sessions	3	65	61	0.76 (-0.33 to 1.85)	0.00	0.17	1.36	100.00
	Length	3	65	61	0.76 (-0.33 to 1.85)	0.00	0.17	1.36	100.00
	Sessions duration	3	65	61	0.76 (-0.33 to 1.85)	0.00	0.17	1.36	100.00
	Survey methods	3	65	61	**0.69 (0.33 to 1.05**)	0.00	0.00	3.72	**-**
Pain	Region	5	111	106	-0.36 (-1.61 to 0.89)	62.68	0.57	-0.57	0.00
	Sessions	5	111	106	-0.36 (-0.80 to 0.09)	36.32	0.12	-1.56	48.15
	Length	5	111	106	-0.16 (-0.71 to 0.39)	60.79	0.56	-0.58	0.00
	Sessions duration	5	111	106	0.18 (-0.23 to 0.59)	0.00	0.40	0.85	0.00
	Survey methods	5	111	106	-0.27 (-0.74 to 0.21)	47.53	0.27	-1.10	18.91
General health status	Region	4	88	84	0.64 (-0.38 to 1.67)	0.00	0.22	1.23	0.00
	Sessions	4	88	84	0.03 (-0.32 to 0.37)	0.00	0.88	0.15	0.00
	Length	4	88	84	0.03 (-0.32 to 0.37)	0.00	0.88	0.15	0.00
	Sessions duration	4	88	84	–	–	–	–	–
	Survey methods	4	88	84	-0.09 (-0.78 to 0.59)	0.00	0.79	-0.26	0.00
SF_36_vitality	Region	3	65	61	0.35 (-0.63 to 1.33)	0.00	0.49	0.70	0.00
	Sessions	3	65	61	-0.35 (-1.33 to 0.63)	0.00	0.49	-0.70	0.00
	Length	3	65	61	0.09 (-0.16 to 0.33)	0.00	0.49	0.70	0.00
	Sessions duration	3	65	61	0.35 (-0.63 to 1.33)	0.00	0.49	0.70	0.00
	Survey methods	3	65	61	–	–	–	–	–
SF_36_social aspects	Region	3	65	61	0.32 (-0.66 to 1.30)	0.00	0.52	0.64	0.00
	Sessions	3	65	61	-0.32 (-1.30 to 0.66)	0.00	0.52	-0.64	0.00
	Length	3	65	61	0.08 (-0.16 to 0.33)	0.00	0.52	0.64	0.00
	Sessions duration	3	65	61	0.32 (-0.66 to 1.30)	0.00	0.52	0.64	0.00
	Survey methods	3	65	61	–	–	–	–	–
ESSDAI	Region	3	65	61	-0.09 (-0.41 to 0.22)	0.00	0.58	-0.56	–
	Sessions	3	65	61	0.16 (-0.54 to 0.86)	0.00	0.66	0.44	0.00
	Length	3	65	61	-0.09 (-0.41 to 0.22)	0.00	0.58	-0.56	–
	Sessions duration	3	65	61	0.02 (-0.56 to 0.60)	0.00	0.95	0.07	–
	Survey methods	3	65	61	-0.09 (-0.41 to 0.23)	0.00	0.58	-0.56	–
Fatigue	Region	4	71	68	-0.64 (-2.98 to 1.70)	90.46	0.59	-0.54	0.00
	Sessions	4	71	68	-0.59 (-3.27 to 2.08)	87.22	0.66	-0.44	0.00
	Length	4	71	68	-0.64 (-2.98 to 1.70)	90.46	0.59	-0.54	0.00
	Sessions duration	4	71	68	-1.39 (-4.52 to 1.75)	85.15	0.39	-0.87	0.00
	Survey methods	4	71	68	-0.59 (-1.37 to 0.18)	81.11	0.13	-1.50	33.80
Mental health	Region	8	162	154	0.07 (-0.69 to 0.82)	49.21	0.86	0.17	0.00
	Sessions	8	162	154	-0.23 (-0.84 to 0.37)	36.59	0.45	-0.76	2.62
	Length	8	162	154	-0.02 (-0.21 to 0.17)	49.21	0.86	-0.17	0.00
	Sessions duration	8	162	154	0.55 (-1.48 to 2.58)	73.87	0.59	0.53	0.00
	Survey methods	8	162	154	0.02 (-0.26 to 0.31)	50.83	0.87	0.16	0.00

CG, control group; CI, confidence interval; EG, exercise group; SF_36_vitality, 36 item short form health survey vitality; SF_36_social aspects, 36 item short form health survey social aspects.

**Table 4:** Subgroup analyses for various outcomes.

Subgroup analysis	Subgroup	Number of Studies	EG	CG	SMD, 95% CI	I^2^(%)	P_heterogeneity_	Z-Value
Subgroup analysis based on cardiopulmonary function								
Region	Over all	5	115	114	**0.59 (0.20 to 0.99)**	51	0.08	2.94
	Developing	4	106	104	**0.52 (0.10 to 0.94)**	56	0.08	
	Developed	1	9	10	**1.13 (0.15 to 2.12)**	–	–	
Sessions (times per week)	Over all	5	115	114	**0.59 (0.20 to 0.99)**	51	0.08	2.94
	2	2	60	60	**0.78 (0.10 to 1.46)**	70	0.07	
	3	3	55	54	0.38 (-0.01 to 0.76)	31	0.23	
Length (weeks)	Over all	5	115	114	**0.59 (0.20 to 0.99)**	51	0.08	2.94
	28	2	60	60	**0.78 (0.10 to 1.46)**	70	0.07	
	16	2	46	44	0.24 (-0.17 to 0.66)	0	0.62	
	12	1	9	10	**1.13 (0.15 to 2.12)**	–	–	
Sessions duration (min)	Over all	5	115	114	**0.59 (0.20 to 0.99)**	51	0.08	2.94
	45	3	69	70	**0.85 (0.33 to 1.36)**	47	0.15	
	35	2	46	44	0.24 (-0.17 to 0.66)	0	0.62	
Survey methods	Over all	5	115	114	**0.59 (0.20 to 0.99)**	51	0.08	2.94
	VO_2_ max	3	62	62	**0.84 (0.27 to 1.40)**	51	0.13	
	HR peak	2	53	52	0.31 (-0.08 to 0.69)	0	0.45	
Subgroup analysis based on functional capacity								
Region	Over all	3	65	61	**0.69 (0.33 to 1.05)**	0	0.39	3.72
	Developing	2	56	51	**0.59 (0.20 to 0.98)**	0	0.94	
	Developed	1	9	10	**1.35 (0.33 to 2.37)**	–	–	
Sessions (times per week)	Over all	3	65	61	**0.69 (0.33 to 1.05)**	0	0.39	3.72
	2	2	56	51	**0.59 (0.20 to 0.98)**	0	0.94	
	3	1	9	10	**1.35 (0.33 to 2.37)**	–	–	
Length (weeks)	Over all	3	65	61	**0.69 (0.33 to 1.05)**	0	0.39	3.72
	16	2	56	51	**0.59 (0.20 to 0.98)**	0	0.94	
	12	1	9	10	**1.35 (0.33 to 2.37)**	–	–	
Sessions duration (min)	Over all	3	65	61	**0.69 (0.33 to 1.05)**	0	0.39	3.72
	NR	2	56	51	**0.59 (0.20 to 0.98)**	0	0.94	
	45	1	9	10	**1.35 (0.33 to 2.37)**	–	–	
Survey methods	Over all	3	65	61	**0.69 (0.33 to 1.05)**	0	0.39	3.72
	functional capacity	3	65	61	**0.69 (0.33 to 1.05)**	0	0.39	
Subgroup analysis based on pain								
Region	Over all	5	111	106	0.32 (-0.09 to 0.72)	53	0.07	1.53
	Developing	4	102	96	0.36 (-0.10 to 0.83)	62	0.05	
	Developed	1	9	10	0.00 (-0.90 to 0.90)	–	–	
Sessions (times per week)	Over all	5	111	106	0.32 (-0.09 to 0.72)	53	0.07	1.53
	2	2	56	51	**0.72 (0.33 to 1.11)**	0	0.36	
	3	2	32	32	-0.14 (-0.63 to 0.36)	0	0.72	
	5	1	23	23	0.16 (-0.42 to 0.74)	–	–	
Length (weeks)	Over all	5	111	106	0.32 (-0.09 to 0.72)	53	0.07	1.53
	16	2	56	51	**0.72 (0.33 to 1.11)**	0	0.36	
	16	1	23	22	-0.19 (-0.78 to 0.39)	–	–	
	12	1	9	10	0.00 (-0.90 to 0.90)	–	–	
	8	1	23	23	0.16 (-0.42 to 0.74)	–	–	
Sessions duration (min)	Over all	5	111	106	0.32 (-0.09 to 0.72)	53	0.07	1.53
	NR	2	56	51	**0.72 (0.33 to 1.11)**	0	0.36	
	35	1	23	22	-0.19 (-0.78 to 0.39)	–	–	
	45	1	9	10	0.00 (-0.90 to 0.90)	–	–	
	30	1	23	23	0.16 (-0.42 to 0.74)	–	–	
Survey methods	Over all	5	111	106	0.32 (-0.09 to 0.72)	53	0.07	1.53
	SF_36_pain	3	65	61	**0.59 (0.17 to 1.01)**	32	0.23	
	ESSPRI_pain	1	23	22	-0.19 (-0.78 to 0.39)	–	–	
	VAS	1	23	23	0.16 (-0.42 to 0.74)	–	–	
Subgroup analysis based on general health status								
Region	Over all	4	88	84	**0.46 (0.15 to 0.76)**	0	0.68	2.93
	Developing	3	79	74	**0.39 (0.07 to 0.71)**	0	0.99	
	Developed	1	9	10	**1.03 (0.06 to 2.01)**	–	–	
Sessions (times per week)	Over all	4	88	84	**0.46 (0.15 to 0.76)**	0	0.68	2.93
	2	2	56	51	**0.39 (0.01 to 0.78)**	0	0.92	
	3	1	9	10	**1.03 (0.06 to 2.01)**	–	–	
	5	1	23	23	0.39 (-0.20 to 0.97)	–	–	
Length (weeks)	Over all	4	88	84	**0.46 (0.15 to 0.76)**	0	0.68	2.93
	16	2	56	51	**0.39 (0.01 to 0.78)**	0	0.92	
	12	1	9	10	**1.03** (**0.06 to 2.01)**	–	–	
	8	1	23	23	0.39 (-0.20 to 0.97)	–	–	
Sessions duration (min)	Over all	4	88	84	**0.46 (0.15 to 0.76)**	0	0.68	2.93
	NR	2	56	51	**0.39 (0.01 to 0.78)**	0	0.92	
	45	1	9	10	**1.03 (0.06 to 2.01)**	–	–	
	30	1	23	23	0.39 (-0.20 to 0.97)	–	–	
Survey methods	Over all	4	88	84	**0.46 (0.15 to 0.76)**	0	0.68	2.93
	SF_36_ general health	3	65	61	**0.48 (0.12 to 0.84)**	0	0.48	
	HAQ	1	23	23	**0.46 (0.15 to 0.76)**	–	–	
Subgroup analysis based on SF_36_vitality								
Region	Over all	3	65	61	**0.51 (0.15 to 0.86)**	0	0.69	2.79
	Developing	2	9	10	**0.56** (**0.17 to 0.95)**	0	0.62	
	Developed	1	56	51	0.21 (-0.69 to 1.12)	–	–	
Sessions (times per week)	Over all	3	65	61	**0.51** (**0.15 to 0.86)**	0	0.69	2.79
	2	2	56	51	**0.56** (**0.17 to 0.95)**	0	0.62	
	3	1	9	10	0.21 (-0.69 to 1.12)	–	–	
Length (weeks)	Over all	3	65	61	**0.51** (**0.15 to 0.86)**	0	0.69	2.79
	16	2	56	51	**0.56 (0.17 to 0.95)**	0	0.62	
	12	1	9	10	0.21 (-0.69 to 1.12)	–	–	
Sessions duration (min)	Over all	3	65	61	**0.51** (**0.15 to 0.86)**	0	0.69	2.79
	NR	2	56	51	**0.56** (**0.17 to 0.95)**	0	0.62	
	45	1	9	10	0.21 (-0.69 to 1.12)	–	–	
Survey methods	Over all	3	65	61	**0.51 (0.15 to 0.86)**	0	0.69	2.79
	SF_36_vitality	3	65	61	**0.51 (0.15 to 0.86)**	0	0.69	2.79
Subgroup analysis based on SF_36_social aspects								
Region	Over all	3	65	61	0.27 (-0.08 to 0.62)	0	0.81	1.52
	Developing	2	56	51	0.32 (-0.06 to 0.70)	0	0.92	
	Developed	1	9	10	0.00 (-0.90 to 0.90)	–	–	
Sessions (times per week)	Over all	3	65	61	0.27 (-0.08 to 0.62)	0	0.81	1.52
	2	2	56	51	0.32 (-0.06 to 0.70)	0	0.92	
	3	1	9	10	0.00 (-0.90 to 0.90)	–	–	
Length (weeks)	Over all	3	65	61	0.27 (-0.08 to 0.62)	0	0.81	1.52
	16	2	56	51	0.32 (-0.06 to 0.70)	0	0.92	
	12	1	9	10	0.00 (-0.90 to 0.90)	–	–	
Sessions duration (min)	Over all	3	65	61	0.27 (-0.08 to 0.62)	0	0.81	1.52
	NR	2	56	51	0.32 (-0.06 to 0.70)	0	0.92	
	45	1	9	10	0.00 (-0.90 to 0.90)	–	–	
Survey methods	Over all	3	65	61	0.27 (-0.08 to 0.62)	0	0.81	1.52
	SF_36_social aspects	3	65	61	0.27 (-0.08 to 0.62)	0	0.81	
Subgroup analysis based on ESSDAI								
Region	Over all	3	65	61	-0.09 (-0.41 to 0.23)	0	0.88	-0.56
	Developing	3	65	61	-0.09 (-0.41 to 0.23)	0	0.88	
Sessions (times per week)	Over all	3	65	61	-0.09 (-0.41 to 0.23)	0	0.88	-0.56
	2	2	56	51	-0.14 (-0.52 to 0.24)	0	0.81	
	3	1	23	22	0.02 (-0.56 to 0.60)	–	–	
Length (weeks)	Over all	3	65	61	-0.09 (-0.41 to 0.23)	0	0.88	-0.56
	16	3	65	61	-0.09 (-0.41 to 0.23)	0	0.88	
Sessions duration (min)	Over all	3	65	61	-0.09 (-0.41 to 0.23)	0	0.88	-0.56
	NR	2	56	51	-0.14 (-0.52 to 0.24)	0	0.81	
	35	1	23	22	0.02 (-0.56 to 0.60)	–	–	
Survey methods	Over all	3	65	61	-0.09 (-0.41 to 0.23)	0	0.88	-0.56
	ESSDAI	3	65	61	-0.09 (-0.41 to 0.23)	0	0.88	
Subgroup analysis based on fatigue								
Region	Over all	4	71	68	-0.57 (-1.57 to 0.44)	85	<0.01	-1.11
	Developing	2	53	48	-0.28 (-1.73 to 1.17)	92	<0.01	
	Developed	2	18	20	-0.92 (-2.79 to 0.95)	85	<0.01	
Sessions (times per week)	Over all	4	71	68	-0.57 (-1.57 to 0.44)	85	<0.01	-1.11
	3	3	41	42	-0.42 (-1.80 to 0.96)	85	<0.01	
	2	1	30	26	**-1.01 (-1.57 to -0.45**)	–	–	
Length (weeks)	Over all	4	71	68	-0.57 (-1.57 to 0.44)	85	<0.01	-1.11
	16	1	23	22	0.46 (-0.13 to 1.06)	–	–	
	16	1	30	26	**-1.01 (-1.57 to -0.45**)	–	–	
	12	2	18	20	-0.92 (-2.79 to 0.95)	85	<0.01	
Sessions duration (min)	Over all	4	71	68	-0.57 (-1.57 to 0.44)	85	<0.01	-1.11
	35	1	23	22	0.46 (-0.13 to 1.06)	–	–	
	NR	1	30	26	**-1.01 (-1.57 to -0.45**)	–	–	
	45	2	18	20	-0.92 (-2.79 to 0.95)	85	<0.01	
Survey methods	Over all	4	71	68	-0.57 (-1.57 to 0.44)	85	<0.01	-1.11
	FACIT_fatigue	1	23	22	0.46 (-0.13 to 1.06)	–	–	
	ESSPRI_fatigue	1	30	26	**-1.01 (-1.57 to -0.45**)	–	–	
	Prof_fatigue	1	9	10	0.00 (-0.90 to 0.90)	–	–	
	RPE	1	9	10	**-1.91 (-3.04 to -0.78**)	–	–	
Subgroup analysis based on mental health								
Region	Over all	8	162	154	**0.42 (0.13 to 0.72**)	45	0.08	2.80
	Developing	5	135	124	**0.41 (0.12 to 0.70**)	29	0.23	
	Developed	3	27	30	0.53 (-0.55 to 1.62)	72	0.03	
Sessions (times per week)	Over all	8	162	154	**0.42 (0.13 to 0.72**)	45	0.08	2.80
	2	4	112	102	**0.5 (0.23 to 0.78**)	5	0.37	
	3	4	50	52	0.35 (-0.40 to 1.11)	64	0.04	
Length (weeks)	Over all	8	162	154	**0.42 (0.13 to 0.72**)	45	0.08	2.80
	16	4	112	102	**0.5 (0.23 to 0.78**)	5	0.37	
	16	1	23	22	-0.02 (-0.60 to 0.57)	–	–	
	12	3	27	30	0.53 (-0.55 to 1.62)	72	0.03	
Sessions duration (min)	Over all	8	162	154	**0.42 (0.13 to 0.72**)	45	0.08	2.80
	NR	4	112	102	**0.5 (0.23 to 0.78**)	5	0.37	
	35	1	23	22	-0.02 (-0.60 to 0.57)	–	–	
	45	3	27	30	0.53 (-0.55 to 1.62)	72	0.03	
Survey methods	Over all	8	162	154	**0.42 (0.13 to 0.72**)	45	0.08	2.80
	SF_36_emotional	2	56	51	**0.74 (0.34 to 1.13**)	0	0.69	
	SF_36_mental health	3	65	61	0.24 (-0.11 to 0.59)	0	0.7	
	BDI	1	23	22	-0.02 (-0.60 to 0.57)	–	–	
	HADS_Anxiety	1	9	10	0.00 (-0.90 to 0.90)	–	–	
	HADS_Depression	1	9	10	**1.72 (0.63 to 2.81**)	–	–	

BDI, beck depression inventory; CG, control group; CI, confidence interval; EG, exercise group; ESSDAI, EULAR Sjögren's syndrome disease activity index; ESSPRI_fatigue, EULAR Sjögren’s syndrome patient reported index fatigue; ESSPRI_pain, EULAR Sjögren’s syndrome patient reported index pain; FACIT_fatigue, functional assessment of chronic illness therapy fatigue; HADS, hospital anxiety and depression scale; HAQ, health assessment questionnaire; HR peak, heart rate peak; NR, not report; Prof_fatigue, profile of fatigue; RPE, ratings of perceived exertion; SF_36_emotional, 36 item short form health survey emotional; SF_36_mental health, 36 item short form health survey mental health; SF_36_pain, 36 item short form health survey pain; SF_36_social aspects, 36 item short form health survey social aspects; SF_36_vitality, 36 item short form health survey vitality; SMD, standardised mean difference; VAS, visual analogue scale; VO2 max, maximal oxygen uptake.

#### 3.4.2 Functional capacity

Three studies reported functional capacity (39–41), involving a total of 126 individuals. Meta-analysis results demonstrated that physical activity significantly improved functional capacity in individuals with SjD (SMD 0.69 [95% CI 0.33 to 1.05]; Q = 1.86, df = 2, P = 0.395, I^2^ = 0%) (**Table 2**). Funnel plot analysis revealed symmetry indicated no significant publication bias (*Pegger* = 0.08) (**Supplementary Figure S6** in **Appendix 4**). Univariate regression analysis identified survey methods as a significant moderator of outcomes, while no significant associations were observed with other covariates (**Table 3**). Subgroup analyses stratified by region, sessions, length, sessions duration, and survey methods demonstrated that all covariates influenced the efficacy of physical activity interventions. Detailed results are presented in **Table 4**.

#### 3.4.3 Pain

Five studies evaluated pain (38–42), encompassing 217 individuals. Meta-analysis indicated no significant effect of physical activity on pain reduction (SMD 0.32 [95% CI -0.09 to 0.72]; Q = 8.56, df = 4, P = 0.073, I^2^ = 53%) (**Table 2**). Funnel plot symmetry suggested no significant publication bias (*Pegger* = 0.59) (**Supplementary Figure S7** in **Appendix 4**). Univariate regression analysis showed no significant associations between covariates and outcomes (**Table 3**). Subgroup analyses of region, sessions, length, session duration, and survey methods revealed that all covariates except region influenced intervention efficacy. Full subgroup results are provided in **Table 4**.

### 3.5 Secondary results

Six studies assessed the effects of physical activity on secondary results in SjD individuals. Significant improvements were observed in general health status (SMD 0.46 [95% CI 0.15 to 0.76]; Q = 1.52, df = 3, P = 0.678, I^2^ = 0%), vitality (SMD 0.51 [95% CI 0.15 to 0.86]; Q = 0.73, df = 2, P = 0.693, I^2^ = 0%), and mental health (SMD 0.42 [95% CI 0.13 to 0.72]; Q = 12.83, df = 7, P = 0.076, I^2^ = 45%). In contrast, no significant effects were detected for social aspects (SMD 0.27 [95% CI -0.08 to 0.62]; Q = 0.42, df = 2, P = 0.809, I^2^ = 0%), ESSDAI (SMD -0.09 [95% CI -0.41 to 0.23]; Q = 0.26, df = 2, P = 0.879, I^2^ = 0%), and fatigue (SMD -0.57 [95% CI -1.57 to 0.44]; Q = 20.4, df = 3, P < 0.001, I^2^ = 85%) (**Table 2**). Univariate regression analyses of these outcomes revealed no significant moderating effects from covariates (**Table 3**). Subgroup analyses for general health status, vitality, mental health, social aspects, ESSDAI, and fatigue are detailed in **Table 4**. Funnel plots for Vitality (*Pegger* = 0.36) (**Supplementary Figure S9** in **Appendix 4**), social aspects (*Pegger* = 0.15) (**Supplementary Figure S10** in **Appendix**), ESSDAI (*Pegger* = 0.6) (**Supplementary Figure S11** in **Appendix 4**), fatigue (*Pegger* = 0.67) (**Supplementary Figure S12** in **Appendix 4**) and mental health (*Pegger=*0.68) (**Supplementary Figure S13** in **Appendix 4**) exhibited symmetry, suggesting low publication bias. general health status funnel plot asymmetry (*Pegger* = 0.02) (**Supplementary Figure S8** in **Appendix 4**) indicated publication bias.

### 3.6 Sensitivity analysis

For outcomes with high heterogeneity, sensitivity analyses were conducted after excluding specific studies (37, 40). Post-exclusion results showed reduced heterogeneity for cardiopulmonary function (SMD 0.59 [95% CI 0.09 to 0.71]) and pain (SMD 0.16 [95% CI -0.20 to 0.52]), with improved stability of intervention effects. These findings highlight the impact of high heterogeneity on outcomes and underscore the importance of sensitivity analyses. Full results are provided in **Appendix 4** (**Supplementary Figures S3**, **S4**).

## 4 Discussion

Our study included six high-quality RCTs, involving 277 female patients with SjD. The findings demonstrate that physical activity interventions significantly improved physical health-related outcomes in SjD individuals, particularly cardiopulmonary function and functional capacity. Additionally, physical activity exhibited favorable effects on general health status, vitality, and mental health compared to controls. However, no significant improvements were observed for pain, social aspects, ESSDAI scores, or fatigue. Regression analyses revealed that survey methods exerted a significant influence on functional capacity outcomes, whereas covariates such as region, sessions, length and sessions duration showed minimal moderating effects. Notably, the GRADE assessment indicated moderate to low certainty of evidence, necessitating cautious interpretation of these results.

Physical activity has garnered significant attention as an effective approach to improving health outcomes in SjD individuals (43), particularly for enhancing cardiopulmonary function and daily living activities (44, 45). This study supports the efficacy of physical activity interventions in improving cardiopulmonary function in SjD individuals, with a statistically significant improvement (SMD 0.59 [95% CI 0.20 to 0.99]). However, substantial heterogeneity was observed in the cardiopulmonary function outcomes. To explore potential sources of heterogeneity, regression analyses were conducted. In addition to intervention characteristics, clinical features of the study populations, such as mean age and disease duration, were also considered as potential moderators of heterogeneity. However, exploratory analyses did not reveal significant associations, suggesting that these factors might not substantially account for between-study variability in the current dataset. Sensitivity analysis, after excluding one study (37), markedly reduced heterogeneity (*I²* = 0%), suggesting that variability in methodological quality among studies may contribute to these discrepancies.

Individuals with SjD are typically older and experience reduced self-care capacity, compounded by disease-related impairments that diminish functional mobility. For instance, declines in muscle endurance, standing balance, grip strength, and joint mobility (46, 47). Evidence from prior meta-analyses supports a positive correlation between physical activity and functional capacity, with significant improvements in walking ability (48), balance, and grip strength (49). Our findings are consistent with these observations. Pain, a key symptom of SjD, is hypothesized to improve with physical activity interventions (43). However, this study did not identify significant pain reduction. Nevertheless, physical activity has been extensively validated in meta-analyses targeting other chronic conditions, including sleep disorders (50), cancer (51), type 2 diabetes (52), obesity (53), and Alzheimer’s disease (54). Although evidence for pain reduction in SjD remains inconclusive, our findings highlight the broader therapeutic potential of physical activity. By demonstrating its benefits for functional capacity in SjD, this study reinforces the role of physical activity as a valuable non-pharmacological intervention for autoimmune disorders.

Reduced physical activity due to pain and prolonged bed rest profoundly impacts mental health, increasing susceptibility to anxiety and depression (55). This study demonstrates that physical activity interventions exert significant positive effects on mental health (SMD 0.42 [95% CI 0.13 to 0.72]), effectively alleviating anxiety and depressive symptoms. vitality, a critical metric for evaluating physical-mental health, daily function, and quality of life, also exhibited significant improvement following physical activity interventions (SMD 0.51 [95% CI 0.15 to 0.86]). Prior research has highlighted that symptoms such as ocular dryness markedly reduce vitality and quality of life in SjD individuals, alongside elevated rates of morbidity and depression (56). Our findings validate this phenomenon and further confirm that physical activity enhances vitality. A meta-analysis similarly underscores aerobic exercise as a means to mitigate fatigue, improve vitality, and enhance quality of life (57). These results emphasize the holistic and integrative role of physical activity in addressing physical, psychological, and social adaptability, beyond disease-specific treatments. Despite the observed benefits for mental health and vitality, physical activity did not significantly alleviate fatigue in SjD individuals (SMD -0.57 [95% CI -1.57 to 0.44]). This lack of significance may stem from fatigue being an intrinsic feature of SjD, closely linked to anhedonia, apathy, reduced activity, social isolation, and depression (58). Consistent with our findings, another meta-analysis concluded that physical activity fails to substantially reduce fatigue in SjD individuals (59). Furthermore, physical activity showed no significant improvements in social aspects or ESSDAI scores, potentially due to the limited number of included studies. Future high-quality research is warranted to explore these outcomes. The optimal physical activity protocol, including subgroup analyses of sessions, length, and sessions duration, revealed that physical activity interventions administered two to three times per week yielded greater health benefits compared to five times per week. Interventions lasting 45-minute sessions outperformed those of 30 or 35 minutes. Additionally, 16 weeks demonstrated optimal efficacy in subgroup analyses. For different outcomes, the optimal intervention duration may vary and should be individually tailored based on patient-specific conditions. Findings from this meta-analysis suggest that resistance and aerobic exercise confer notable health benefits.

### 4.1 Strengths and limitations

This study has several notable strengths. First, it constitutes the systematic review and meta-analysis to evaluate the impact of physical activity interventions on the general health status of individuals with SjD. A comprehensive search across relevant databases was conducted, with rigorous scrutiny of all outcome measures to ensure a robust assessment of physical activity efficacy. Second, regression and subgroup analyses were incorporated to address confounding factors, enhancing the reliability and precision of the results. Finally, the methodological quality of included studies was systematically appraised using validated tools, and evidence-based recommendations were formulated to refine clinical guidelines. These measures collectively strengthen the scientific validity and practical applicability of the conclusions.

However, certain limitations must be acknowledged. First, the limited number of RCTs, small sample sizes, and variability in study quality may affect the precision of the findings. Second, Although we made every effort to include all eligible studies, unfortunately, no studies involving male individuals with SjD were available for analysis. This may limit the representativeness and generalizability of the present meta-analysis to some extent, particularly as the potential impact of sex differences has not been adequately assessed. Furthermore, suboptimal reporting of randomization procedures and substantial heterogeneity in outcome measures introduce uncertainty in data interpretation. Future research should prioritize large-scale, high-quality RCTs with diverse cohorts, including male patients, to reduce bias and enhance external validity. Rigorous study design will yield more robust evidence to facilitate the clinical translation of physical activity interventions in SjD management.

### 4.2 Clinical implications

Physical activity, as a non-pharmacological intervention, offers diverse modalities, customizable protocols, and a high safety profile with minimal adverse effects, thereby improving individual’s adherence in individuals with SjD. In this review, we systematically examined the effects of physical activity on cardiopulmonary function and functional capacity in SjD individuals, revealing that intervention duration emerged as a critical determinant of rehabilitative outcomes. Specifically, short-term regimens showed minimal benefits, whereas sustained adherence to long-term physical activity protocols significantly improved general health status. These findings provide a robust scientific foundation for developing personalized physical activity prescriptions, further underscoring the pivotal role of physical activity in SjD rehabilitation management.

## 5 Conclusion

The findings of this study indicate that physical activity interventions serve as an effective adjunct therapy, significantly improving cardiopulmonary function, functional capacity, general health status, vitality, and mental health in individuals with SjD. Aerobic training and resistance exercise are recommended as the primary modalities due to their feasibility and proven efficacy. However, the moderate-to-low certainty of evidence underscores the need for future research to prioritize the design and implementation of high-quality RCTs.

## Data Availability

The original contributions presented in the study are included in the article/**Supplementary Material**. Further inquiries can be directed to the corresponding author.
