# Supplementary material for: Effects of physical activity on health-related outcomes in Sjögren’s syndrome: a systematic review and meta-analysis of randomized controlled trials
Source: Front Immunol. 2026 Apr 7;17:1753791. doi: 10.3389/fimmu.2026.1753791 (PMC13095831; doi:10.3389/fimmu.2026.1753791)
Supplement: Supplementary file 1 [file Table1.docx]

SUPPLEMENTARY INFORMATION

**Effects of physical activity on health-related outcomes in Sjögren's disease: A**

**systematic review and meta-analysis of randomized controlled trials**

**CONTENTS**

**Appendix 1. Search strategies 1**

**1.1 PubMed 1**

**1.2 Cochrane Library 5**

**1.3 Embase 7**

**1.4 Scopus 9**

**1.5 Web of Science 14**

**1.6 SPORTDiscus 15**

**1.7 ClinicalTrials.gov 15**

**Appendix 2. Risk of bias 17**

**2.1 Risk of bias graph 17**

**2.2 Risk of bias summary 18**

**Appendix 3. GRADE Summary of Evidence 19**

**Appendix 4. Sensitivity Analysis and Publication Bias 20**

**4.1 Sensitivity Analysis 20**

**4.2 Publication Bias 21**

**Appendix 5. PRISMA 2020 Checklist……………………………………………...29**

**Appendix 1. Search strategies**

**1.1 PubMed**

#1 Search:"Sjogren's Syndrome"[Mesh]

#2 ((((((Sjögren's syndrome[Title/Abstract]) ) OR (Sjogrens Syndrome[Title/Abstract])) OR (Syndrome, Sjogren's[Title/Abstract])) OR (Sjogren Syndrome[Title/Abstract])) OR (Sicca Syndrome[Title/Abstract])) OR (Syndrome, Sicca[Title/Abstract])

#3 ("Sjogren's Syndrome"[Mesh]) OR (((((((Sjögren's syndrome[Title/Abstract]) ) OR (Sjogrens Syndrome[Title/Abstract])) OR (Syndrome, Sjogren's[Title/Abstract])) OR (Sjogren Syndrome[Title/Abstract])) OR (Sicca Syndrome[Title/Abstract])) OR (Syndrome, Sicca[Title/Abstract]))

#4 "Exercise"[Mesh]

#5 (((((((((((((((((((((((((exercise[Title/Abstract]) OR (Exercises[Title/Abstract])) OR (Exercise, Physical[Title/Abstract])) OR (Exercises, Physical[Title/Abstract])) OR (Physical Exercise[Title/Abstract])) OR (Physical Exercises[Title/Abstract])) OR (Physical Activity[Title/Abstract])) OR (Activities, Physical[Title/Abstract])) OR (Activity, Physical[Title/Abstract])) OR (Physical Activities[Title/Abstract])) OR (Exercise, Aerobic[Title/Abstract])) OR (Aerobic Exercise[Title/Abstract])) OR (Aerobic Exercises[Title/Abstract])) OR (Exercises, Aerobic[Title/Abstract])) OR (Exercise, Isometric[Title/Abstract])) OR (Exercises, Isometric[Title/Abstract])) OR (Isometric Exercises[Title/Abstract])) OR (Isometric Exercise[Title/Abstract])) OR (Acute Exercise[Title/Abstract])) OR (Acute Exercises[Title/Abstract])) OR (Exercise, Acute[Title/Abstract])) OR (Exercises, Acute[Title/Abstract])) OR (Exercise Training[Title/Abstract])) OR (Exercise Trainings[Title/Abstract])) OR (Training, Exercise[Title/Abstract])) OR (Trainings, Exercise[Title/Abstract])

#6 ("Exercise"[Mesh]) OR ((((((((((((((((((((((((((exercise[Title/Abstract]) OR (Exercises[Title/Abstract])) OR (Exercise, Physical[Title/Abstract])) OR (Exercises, Physical[Title/Abstract])) OR (Physical Exercise[Title/Abstract])) OR (Physical Exercises[Title/Abstract])) OR (Physical Activity[Title/Abstract])) OR (Activities, Physical[Title/Abstract])) OR (Activity, Physical[Title/Abstract])) OR (Physical Activities[Title/Abstract])) OR (Exercise, Aerobic[Title/Abstract])) OR (Aerobic Exercise[Title/Abstract])) OR (Aerobic Exercises[Title/Abstract])) OR (Exercises, Aerobic[Title/Abstract])) OR (Exercise, Isometric[Title/Abstract])) OR (Exercises, Isometric[Title/Abstract])) OR (Isometric Exercises[Title/Abstract])) OR (Isometric Exercise[Title/Abstract])) OR (Acute Exercise[Title/Abstract])) OR (Acute Exercises[Title/Abstract])) OR (Exercise, Acute[Title/Abstract])) OR (Exercises, Acute[Title/Abstract])) OR (Exercise Training[Title/Abstract])) OR (Exercise Trainings[Title/Abstract])) OR (Training, Exercise[Title/Abstract])) OR (Trainings, Exercise[Title/Abstract]))

#7 "Resistance Training"[Mesh]

#8 (((((((((((((((Resistance Training[Title/Abstract]) OR (Training, Resistance[Title/Abstract])) OR (Strength Training[Title/Abstract])) OR (Training, Strength[Title/Abstract])) OR (Weight-Lifting Strengthening Program[Title/Abstract])) OR (Strengthening Programs, Weight-Lifting[Title/Abstract])) OR (Strengthening Program, Weight-Lifting[Title/Abstract])) OR (Weight Lifting Strengthening Program[Title/Abstract])) OR (Weight-Lifting Exercise Program[Title/Abstract])) OR (Exercise Program, Weight-Lifting[Title/Abstract])) OR (Weight-Lifting Exercise Programs[Title/Abstract])) OR (Strengthening Programs, Weight-Bearing[Title/Abstract])) OR (Weight Bearing Strengthening Program[Title/Abstract])) OR (Weight-Bearing Exercise Program[Title/Abstract])) OR (Exercise Program, Weight-Bearing[Title/Abstract])) OR (Weight-Bearing Exercise Programs[Title/Abstract])

#9 ("Resistance Training"[Mesh]) OR ((((((((((((((((Resistance Training[Title/Abstract]) OR (Training, Resistance[Title/Abstract])) OR (Strength Training[Title/Abstract])) OR (Training, Strength[Title/Abstract])) OR (Weight-Lifting Strengthening Program[Title/Abstract])) OR (Strengthening Programs, Weight-Lifting[Title/Abstract])) OR (Strengthening Program, Weight-Lifting[Title/Abstract])) OR (Weight Lifting Strengthening Program[Title/Abstract])) OR (Weight-Lifting Exercise Program[Title/Abstract])) OR (Exercise Program, Weight-Lifting[Title/Abstract])) OR (Weight-Lifting Exercise Programs[Title/Abstract])) OR (Strengthening Programs, Weight-Bearing[Title/Abstract])) OR (Weight Bearing Strengthening Program[Title/Abstract])) OR (Weight-Bearing Exercise Program[Title/Abstract])) OR (Exercise Program, Weight-Bearing[Title/Abstract])) OR (Weight-Bearing Exercise Programs[Title/Abstract]))

#10 "Tai Ji"[Mesh]

#11 (((((((Tai Ji[Title/Abstract]) OR (Tai-ji[Title/Abstract])) OR (Tai Chi[Title/Abstract])) OR (Chi, Tai[Title/Abstract])) OR (Tai Chi Chuan[Title/Abstract])) OR (Taiji[Title/Abstract])) OR (T'ai Chi[Title/Abstract])) OR (Ji Quan, Tai[Title/Abstract])

#12 ("Tai Ji"[Mesh]) OR ((((((((Tai Ji[Title/Abstract]) OR (Tai-ji[Title/Abstract])) OR (Tai Chi[Title/Abstract])) OR (Chi, Tai[Title/Abstract])) OR (Tai Chi Chuan[Title/Abstract])) OR (Taiji[Title/Abstract])) OR (T'ai Chi[Title/Abstract])) OR (Ji Quan, Tai[Title/Abstract]))

#13 "Randomized Controlled Trials as Topic"[Mesh]

#14 (((Randomized Controlled Trials as Topic[Title/Abstract]) OR (Clinical Trials, Randomized[Title/Abstract])) OR (Trials, Randomized Clinical[Title/Abstract])) OR (Controlled Clinical Trials, Randomized[Title/Abstract])

#15 ("Randomized Controlled Trials as Topic"[Mesh]) OR ((((Randomized Controlled Trials as Topic[Title/Abstract]) OR (Clinical Trials, Randomized[Title/Abstract])) OR (Trials, Randomized Clinical[Title/Abstract])) OR (Controlled Clinical Trials, Randomized[Title/Abstract]))

#16 ((((("Sjogren's Syndrome"[Mesh]) OR (((((((Sjögren's syndrome[Title/Abstract]) ) OR (Sjogrens Syndrome[Title/Abstract])) OR (Syndrome, Sjogren's[Title/Abstract])) OR (Sjogren Syndrome[Title/Abstract])) OR (Sicca Syndrome[Title/Abstract])) OR (Syndrome, Sicca[Title/Abstract]))) AND (("Exercise"[Mesh]) OR ((((((((((((((((((((((((((exercise[Title/Abstract]) OR (Exercises[Title/Abstract])) OR (Exercise, Physical[Title/Abstract])) OR (Exercises, Physical[Title/Abstract])) OR (Physical Exercise[Title/Abstract])) OR (Physical Exercises[Title/Abstract])) OR (Physical Activity[Title/Abstract])) OR (Activities, Physical[Title/Abstract])) OR (Activity, Physical[Title/Abstract])) OR (Physical Activities[Title/Abstract])) OR (Exercise, Aerobic[Title/Abstract])) OR (Aerobic Exercise[Title/Abstract])) OR (Aerobic Exercises[Title/Abstract])) OR (Exercises, Aerobic[Title/Abstract])) OR (Exercise, Isometric[Title/Abstract])) OR (Exercises, Isometric[Title/Abstract])) OR (Isometric Exercises[Title/Abstract])) OR (Isometric Exercise[Title/Abstract])) OR (Acute Exercise[Title/Abstract])) OR (Acute Exercises[Title/Abstract])) OR (Exercise, Acute[Title/Abstract])) OR (Exercises, Acute[Title/Abstract])) OR (Exercise Training[Title/Abstract])) OR (Exercise Trainings[Title/Abstract])) OR (Training, Exercise[Title/Abstract])) OR (Trainings, Exercise[Title/Abstract])))) OR (("Resistance Training"[Mesh]) OR ((((((((((((((((Resistance Training[Title/Abstract]) OR (Training, Resistance[Title/Abstract])) OR (Strength Training[Title/Abstract])) OR (Training, Strength[Title/Abstract])) OR (Weight-Lifting Strengthening Program[Title/Abstract])) OR (Strengthening Programs, Weight-Lifting[Title/Abstract])) OR (Strengthening Program, Weight-Lifting[Title/Abstract])) OR (Weight Lifting Strengthening Program[Title/Abstract])) OR (Weight-Lifting Exercise Program[Title/Abstract])) OR (Exercise Program, Weight-Lifting[Title/Abstract])) OR (Weight-Lifting Exercise Programs[Title/Abstract])) OR (Strengthening Programs, Weight-Bearing[Title/Abstract])) OR (Weight Bearing Strengthening Program[Title/Abstract])) OR (Weight-Bearing Exercise Program[Title/Abstract])) OR (Exercise Program, Weight-Bearing[Title/Abstract])) OR (Weight-Bearing Exercise Programs[Title/Abstract])))) OR (("Tai Ji"[Mesh]) OR ((((((((Tai Ji[Title/Abstract]) OR (Tai-ji[Title/Abstract])) OR (Tai Chi[Title/Abstract])) OR (Chi, Tai[Title/Abstract])) OR (Tai Chi Chuan[Title/Abstract])) OR (Taiji[Title/Abstract])) OR (T'ai Chi[Title/Abstract])) OR (Ji Quan, Tai[Title/Abstract])))) AND (("Randomized Controlled Trials as Topic"[Mesh]) OR ((((Randomized Controlled Trials as Topic[Title/Abstract]) OR (Clinical Trials, Randomized[Title/Abstract])) OR (Trials, Randomized Clinical[Title/Abstract])) OR (Controlled Clinical Trials, Randomized[Title/Abstract])))

**1.2 Cochrane Library**

#1 Sjogren's Syndrome

#2 (Sjogren's Syndrome):ti,ab,kw OR (Sjogren Syndrome):ti,ab,kw OR (Syndrome, Sjogren's):ti,ab,kw OR (Sjogrens Syndrome):ti,ab,kw OR (Syndrome, Sicca):ti,ab,kw

#3 #1OR#2

#4 (Exercise):ti,ab,kw OR (Isometric Exercise):ti,ab,kw OR (Exercise, Isometric):ti,ab,kw OR (Exercises, Isometric):ti,ab,kw OR (Isometric Exercises):ti,ab,kw

#5 (Exercise Trainings):ti,ab,kw OR (Training, Exercise):ti,ab,kw OR (Exercise Training):ti,ab,kw OR (Trainings, Exercise):ti,ab,kw OR (Physical Exercises):ti,ab,kw

#6 (Exercises, Physical):ti,ab,kw OR (Physical Activity):ti,ab,kw OR (Activity, Physical):ti,ab,kw OR (Exercise, Physical):ti,ab,kw OR (Exercises):ti,ab,kw

#7 (Activities, Physical):ti,ab,kw OR (Physical Exercise):ti,ab,kw OR (Physical Activities):ti,ab,kw OR (Exercise, Aerobic):ti,ab,kw OR (Aerobic Exercise):ti,ab,kw

#8 (Exercises, Aerobic):ti,ab,kw OR (Aerobic Exercises):ti,ab,kw OR (Acute Exercises):ti,ab,kw OR (Exercise, Acute):ti,ab,kw OR (Acute Exercise):ti,ab,kw

#9 #4 OR #5 OR #6 OR #7 OR #8

#10 (Taijiquan):ti,ab,kw OR (Tai Ji Quan):ti,ab,kw OR (Tai-ji):ti,ab,kw OR (Tai Chi):ti,ab,kw OR (Quan, Tai Ji):ti,ab,kw

#11 (Resistance Training):ti,ab,kw OR (Weight-Lifting Strengthening Programs):ti,ab,kw OR (Strengthening Programs, Weight-Lifting):ti,ab,kw OR (Weight Lifting Exercise Program):ti,ab,kw OR (Weight-Lifting Exercise Program):ti,ab,kw

#12 (Exercise Programs, Weight-Lifting):ti,ab,kw OR (Weight-Lifting Strengthening Program):ti,ab,kw OR (Weight-Lifting Exercise Programs):ti,ab,kw OR (Weight Lifting Strengthening Program):ti,ab,kw OR (Exercise Program, Weight-Lifting):ti,ab,kw

#13 (Strengthening Program, Weight-Lifting):ti,ab,kw OR (Strengthening Program, Weight-Bearing):ti,ab,kw OR (Weight-Bearing Exercise Programs):ti,ab,kw OR (Exercise Programs, Weight-Bearing):ti,ab,kw OR (Weight-Bearing Strengthening Program):ti,ab,kw

#14 (Exercise Program, Weight-Bearing):ti,ab,kw OR (Weight Bearing Exercise Program):ti,ab,kw OR (Weight Bearing Strengthening Program):ti,ab,kw OR (Weight-Bearing Exercise Program):ti,ab,kw OR (Strengthening Programs, Weight-Bearing):ti,ab,kw

#15 (Weight-Bearing Strengthening Programs):ti,ab,kw OR (Strength Training):ti,ab,kw OR (Training, Strength):ti,ab,kw OR (Training, Resistance):ti,ab,kw

#16 #10 OR #11 OR #12 OR #13 OR #14 OR #15

#17 (Random Allocation):ti,ab,kw OR (Allocation, Random):ti,ab,kw OR (Randomization):ti,ab,kw

#18 #3 AND #9 OR #16 AND #17

**1.3 Embase**

#1 'sjoegren syndrome'/exp

#2 sjogrens syndrome OR (sjogrens AND ('syndrome'/exp OR syndrome)) OR 'dacryosialoadenopathia atrophicans':ab,ti OR 'dyssecretosis, mucoserous':ab,ti OR 'gougerot houwer sjoegren syndrome':ab,ti OR 'gougerot sjoegren disease':ab,ti OR 'mikulicz gougerot sjoegren syndrome':ab,ti OR 'mucoserous dyssecretosis':ab,ti OR 'sialosis, rheumatic':ab,ti OR 'sjogren disease':ab,ti OR xerodermosteosis:ab,ti OR 'sjoegren syndrome':ab,ti

#3 #1 OR #2

#4 'exercise'/exp

#5 'exercise'/exp OR exercise OR 'biometric exercise':ab,ti OR effort:ab,ti OR 'exercise capacity':ab,ti OR 'exercise performance':ab,ti OR 'exercise training':ab,ti OR exertion:ab,ti OR 'fitness training':ab,ti OR 'physical conditioning, human':ab,ti OR 'physical effort':ab,ti OR 'physical exertion':ab,ti OR 'physical work-out':ab,ti

#6 #4 OR #5

#7 'physical activity'/exp

#8 'physical activity'/exp OR 'physical activity' OR (physical AND ('activity'/exp OR activity)) OR 'activity, physical':ab,ti OR 'physical activity':ab,ti

#9 #7 OR #8

#10 'training'/exp

#11 'training'/exp OR training OR 'army training':ab,ti OR 'athletic training':ab,ti OR 'athletic training program':ab,ti OR detraining:ab,ti OR 'military training':ab,ti OR 'physical training':ab,ti OR 'sport specific training':ab,ti OR 'technical training':ab,ti OR 'training, physical':ab,ti

#12 #10 OR #11

#13 'aerobic exercise'/exp

#14 'aerobic exercise'/exp OR 'aerobic exercise' OR (aerobic AND ('exercise'/exp OR exercise)) OR 'aerobic dance':ab,ti OR aerobics:ab,ti OR 'aerobics exercise':ab,ti OR 'dancing, aerobic':ab,ti OR 'low impact aerobic exercise':ab,ti OR 'step aerobics':ab,ti

#15 #13 OR #14

#16 'resistance training'/exp

#17 'resistance training'/exp OR 'resistance training' OR (('resistance'/exp OR resistance) AND ('training'/exp OR training)) OR 'resistance exercise':ab,ti OR 'resistance exercise training':ab,ti OR 'resistance-type training':ab,ti OR 'strength training':ab,ti OR 'strength-type training':ab,ti OR 'resistance training':ab,ti

#18 #16 OR #17

#19 'qigong'/exp

#20 'qigong'/exp OR qigong OR 'chi kung':ab,ti OR chigung:ab,ti OR 'qi gong':ab,ti

#21 #19 OR #20

#22 'tai chi'/exp

#23 'tai chi chuan'/exp OR 'tai chi chuan' OR (tai AND chi AND chuan) OR 'tai ji':ab,ti OR 'taiji quan':ab,ti OR taijiquan:ab,ti OR 'tai chi':ab,ti

#24 #22 OR #23

#25 'randomized controlled trial'/exp

#26 'controlled trial, randomized'/exp OR 'controlled trial, randomized' OR (controlled AND trial, AND randomized) OR 'randomised controlled study':ab,ti OR 'randomised controlled trial':ab,ti OR 'randomized controlled study':ab,ti OR 'trial, randomized controlled':ab,ti OR 'randomized controlled trial':ab,ti

#27 #25 OR #26

#28 #6 OR #9 OR #12 OR #15 OR #18 OR #21 OR #24

#29 #3 AND #27 AND #28

**1.4 Scopus**

#1 ( TITLE-ABS-KEY ( sjogren's AND syndrome ) OR TITLE-ABS-KEY ( sjogrens AND syndrome ) OR TITLE-ABS-KEY ( syndrome, AND sjogren's ) OR TITLE-ABS-KEY ( sjogren AND syndrome ) OR TITLE-ABS-KEY ( sicca AND syndrome ) OR TITLE-ABS-KEY ( syndrome, AND sicca ) )

#2 ( TITLE-ABS-KEY ( exercise ) OR TITLE-ABS-KEY ( exercise, AND physical ) OR TITLE-ABS-KEY ( exercises, AND physical ) OR TITLE-ABS-KEY ( physical AND exercise ) OR TITLE-ABS-KEY ( physical AND activity ) OR TITLE-ABS-KEY ( activities, AND physical ) OR TITLE-ABS-KEY ( activity, AND physical ) OR TITLE-ABS-KEY ( physical AND activities ) OR TITLE-ABS-KEY ( exercise, AND aerobic ) OR TITLE-ABS-KEY ( aerobic AND exercise ) OR TITLE-ABS-KEY ( aerobic AND exercises ) OR TITLE-ABS-KEY ( exercises, AND aerobic ) OR TITLE-ABS-KEY ( exercise, AND isometric ) OR TITLE-ABS-KEY ( exercises, AND isometric ) OR TITLE-ABS-KEY ( isometric AND exercises ) OR TITLE-ABS-KEY ( isometric AND exercise ) OR TITLE-ABS-KEY ( acute AND exercise ) OR TITLE-ABS-KEY ( acute AND exercises ) OR TITLE-ABS-KEY ( exercise AND training ) OR TITLE-ABS-KEY ( trainings, AND exercise ) )

#3 ( TITLE-ABS-KEY ( resistance AND training ) OR TITLE-ABS-KEY ( strength AND training ) OR TITLE-ABS-KEY ( weight-lifting AND strengthening AND program ) OR TITLE-ABS-KEY ( strengthening AND programs, AND weight-lifting ) OR TITLE-ABS-KEY ( weight AND lifting AND strengthening AND program ) OR TITLE-ABS-KEY ( weight-lifting AND strengthening AND programs ) OR TITLE-ABS-KEY ( weight-lifting AND exercise AND program ) OR TITLE-ABS-KEY ( exercise AND programs, AND weight-lifting ) OR TITLE-ABS-KEY ( weight AND lifting AND exercise AND program ) OR TITLE-ABS-KEY ( weight-bearing AND strengthening AND program ) OR TITLE-ABS-KEY ( strengthening AND programs, AND weight-bearing ) OR TITLE-ABS-KEY ( weight AND bearing AND strengthening AND program ) OR TITLE-ABS-KEY ( weight-bearing AND exercise AND program ) OR TITLE-ABS-KEY ( exercise AND programs, AND weight-bearing ) OR TITLE-ABS-KEY ( weight AND bearing AND exercise AND program ) )

#4 ( TITLE-ABS-KEY ( qigong ) OR TITLE-ABS-KEY ( ch'i AND kung ) OR TITLE-ABS-KEY ( qi AND gong ) )

#5 ( TITLE-ABS-KEY ( tai AND ji ) OR TITLE-ABS-KEY ( tai-ji ) OR TITLE-ABS-KEY ( tai AND chi ) OR TITLE-ABS-KEY ( tai AND chi AND chuan ) OR TITLE-ABS-KEY ( taiji ) OR TITLE-ABS-KEY ( taijiquan ) OR TITLE-ABS-KEY ( t'ai AND chi ) OR TITLE-ABS-KEY ( ji AND quan, AND tai ) OR TITLE-ABS-KEY ( quan, AND tai AND ji ) )

#6 ( TITLE-ABS-KEY ( randomized AND controlled AND trials AND as AND topic ) OR TITLE-ABS-KEY ( clinical AND trials, AND randomized ) OR TITLE-ABS-KEY ( trials, AND randomized AND clinical ) OR TITLE-ABS-KEY ( controlled AND clinical AND trials, AND randomized ) )

#7 ( ( TITLE-ABS-KEY ( exercise ) OR TITLE-ABS-KEY ( exercise, AND physical ) OR TITLE-ABS-KEY ( exercises, AND physical ) OR TITLE-ABS-KEY ( physical AND exercise ) OR TITLE-ABS-KEY ( physical AND activity ) OR TITLE-ABS-KEY ( activities, AND physical ) OR TITLE-ABS-KEY ( activity, AND physical ) OR TITLE-ABS-KEY ( physical AND activities ) OR TITLE-ABS-KEY ( exercise, AND aerobic ) OR TITLE-ABS-KEY ( aerobic AND exercise ) OR TITLE-ABS-KEY ( aerobic AND exercises ) OR TITLE-ABS-KEY ( exercises, AND aerobic ) OR TITLE-ABS-KEY ( exercise, AND isometric ) OR TITLE-ABS-KEY ( exercises, AND isometric ) OR TITLE-ABS-KEY ( isometric AND exercises ) OR TITLE-ABS-KEY ( isometric AND exercise ) OR TITLE-ABS-KEY ( acute AND exercise ) OR TITLE-ABS-KEY ( acute AND exercises ) OR TITLE-ABS-KEY ( exercise AND training ) OR TITLE-ABS-KEY ( trainings, AND exercise ) ) ) OR ( ( TITLE-ABS-KEY ( resistance AND training ) OR TITLE-ABS-KEY ( strength AND training ) OR TITLE-ABS-KEY ( weight-lifting AND strengthening AND program ) OR TITLE-ABS-KEY ( strengthening AND programs, AND weight-lifting ) OR TITLE-ABS-KEY ( weight AND lifting AND strengthening AND program ) OR TITLE-ABS-KEY ( weight-lifting AND strengthening AND programs ) OR TITLE-ABS-KEY ( weight-lifting AND exercise AND program ) OR TITLE-ABS-KEY ( exercise AND programs, AND weight-lifting ) OR TITLE-ABS-KEY ( weight AND lifting AND exercise AND program ) OR TITLE-ABS-KEY ( weight-bearing AND strengthening AND program ) OR TITLE-ABS-KEY ( strengthening AND programs, AND weight-bearing ) OR TITLE-ABS-KEY ( weight AND bearing AND strengthening AND program ) OR TITLE-ABS-KEY ( weight-bearing AND exercise AND program ) OR TITLE-ABS-KEY ( exercise AND programs, AND weight-bearing ) OR TITLE-ABS-KEY ( weight AND bearing AND exercise AND program ) ) ) OR ( ( TITLE-ABS-KEY ( qigong ) OR TITLE-ABS-KEY ( ch'i AND kung ) OR TITLE-ABS-KEY ( qi AND gong ) ) ) OR ( ( TITLE-ABS-KEY ( tai AND ji ) OR TITLE-ABS-KEY ( tai-ji ) OR TITLE-ABS-KEY ( tai AND chi ) OR TITLE-ABS-KEY ( tai AND chi AND chuan ) OR TITLE-ABS-KEY ( taiji ) OR TITLE-ABS-KEY ( taijiquan ) OR TITLE-ABS-KEY ( t'ai AND chi ) OR TITLE-ABS-KEY ( ji AND quan, AND tai ) OR TITLE-ABS-KEY ( quan, AND tai AND ji ) ) )

#8 ( ( TITLE-ABS-KEY ( sjogren's AND syndrome ) OR TITLE-ABS-KEY ( sjogrens AND syndrome ) OR TITLE-ABS-KEY ( syndrome, AND sjogren's ) OR TITLE-ABS-KEY ( sjogren AND syndrome ) OR TITLE-ABS-KEY ( sicca AND syndrome ) OR TITLE-ABS-KEY ( syndrome, AND sicca ) ) ) AND ( ( TITLE-ABS-KEY ( randomized AND controlled AND trials AND as AND topic ) OR TITLE-ABS-KEY ( clinical AND trials, AND randomized ) OR TITLE-ABS-KEY ( trials, AND randomized AND clinical ) OR TITLE-ABS-KEY ( controlled AND clinical AND trials, AND randomized ) ) ) AND ( ( ( TITLE-ABS-KEY ( exercise ) OR TITLE-ABS-KEY ( exercise, AND physical ) OR TITLE-ABS-KEY ( exercises, AND physical ) OR TITLE-ABS-KEY ( physical AND exercise ) OR TITLE-ABS-KEY ( physical AND activity ) OR TITLE-ABS-KEY ( activities, AND physical ) OR TITLE-ABS-KEY ( activity, AND physical ) OR TITLE-ABS-KEY ( physical AND activities ) OR TITLE-ABS-KEY ( exercise, AND aerobic ) OR TITLE-ABS-KEY ( aerobic AND exercise ) OR TITLE-ABS-KEY ( aerobic AND exercises ) OR TITLE-ABS-KEY ( exercises, AND aerobic ) OR TITLE-ABS-KEY ( exercise, AND isometric ) OR TITLE-ABS-KEY ( exercises, AND isometric ) OR TITLE-ABS-KEY ( isometric AND exercises ) OR TITLE-ABS-KEY ( isometric AND exercise ) OR TITLE-ABS-KEY ( acute AND exercise ) OR TITLE-ABS-KEY ( acute AND exercises ) OR TITLE-ABS-KEY ( exercise AND training ) OR TITLE-ABS-KEY ( trainings, AND exercise ) ) ) OR ( ( TITLE-ABS-KEY ( resistance AND training ) OR TITLE-ABS-KEY ( strength AND training ) OR TITLE-ABS-KEY ( weight-lifting AND strengthening AND program ) OR TITLE-ABS-KEY ( strengthening AND programs, AND weight-lifting ) OR TITLE-ABS-KEY ( weight AND lifting AND strengthening AND program ) OR TITLE-ABS-KEY ( weight-lifting AND strengthening AND programs ) OR TITLE-ABS-KEY ( weight-lifting AND exercise AND program ) OR TITLE-ABS-KEY ( exercise AND programs, AND weight-lifting ) OR TITLE-ABS-KEY ( weight AND lifting AND exercise AND program ) OR TITLE-ABS-KEY ( weight-bearing AND strengthening AND program ) OR TITLE-ABS-KEY ( strengthening AND programs, AND weight-bearing ) OR TITLE-ABS-KEY ( weight AND bearing AND strengthening AND program ) OR TITLE-ABS-KEY ( weight-bearing AND exercise AND program ) OR TITLE-ABS-KEY ( exercise AND programs, AND weight-bearing ) OR TITLE-ABS-KEY ( weight AND bearing AND exercise AND program ) ) ) OR ( ( TITLE-ABS-KEY ( qigong ) OR TITLE-ABS-KEY ( ch'i AND kung ) OR TITLE-ABS-KEY ( qi AND gong ) ) ) OR ( ( TITLE-ABS-KEY ( tai AND ji ) OR TITLE-ABS-KEY ( tai-ji ) OR TITLE-ABS-KEY ( tai AND chi ) OR TITLE-ABS-KEY ( tai AND chi AND chuan ) OR TITLE-ABS-KEY ( taiji ) OR TITLE-ABS-KEY ( taijiquan ) OR TITLE-ABS-KEY ( t'ai AND chi ) OR TITLE-ABS-KEY ( ji AND quan, AND tai ) OR TITLE-ABS-KEY ( quan, AND tai AND ji ) ) ) )

**1.5 Web of Science**

#1 TS=(Sjögren's syndrome OR Sjogrens Syndrome OR Syndrome, Sjogren's OR Sjogren Syndrome OR Sicca Syndrome OR Syndrome, Sicca)

#2 TS=(Exercise OR Exercises OR Physical Activity OR Activities, Physical OR Activity, Physical OR Physical Activities OR Exercise, Physical OR Exercises, Physical OR Physical Exercise OR Physical Exercises OR Exercise, Acute OR Exercises, Acute OR Exercise, Isometric OR Exercises, Isometric OR Isometric Exercises OR Isometric Exercise OR Exercise, Aerobic OR Aerobic Exercise OR Aerobic Exercises OR Exercises, Aerobic OR Exercise Training OR Exercise Trainings OR Training, Exercise OR Trainings, Exercise)

#3 TS=(Resistance Training OR Training, Resistance OR Strength Training OR Training, Strength OR Weight-Lifting Strengthening Program OR Strengthening Programs, Weight-Lifting OR Strengthening Program, Weight-Lifting OR Weight Lifting Strengthening Program OR Weight-Lifting Exercise Program OR Exercise Program, Weight-Lifting OR Weight-Lifting Exercise Programs OR Strengthening Programs, Weight-Bearing OR Weight Bearing Strengthening Program OR Weight-Bearing Exercise Program OR Exercise Program, Weight-Bearing OR Weight-Bearing Exercise Programs)

#4 TS=(Tai Ji OR Tai-ji OR Tai Chi OR Chi, Tai OR Tai Chi Chuan OR Taiji OR T'ai Chi OR Ji Quan, Tai)

#5 TS=(randomized controlled trial OR randomized OR placebo)

#6 #2 OR #3 OR #4

#7 #1 AND #5 AND #6

**1.6 SPORTDiscus**

S1 AB Sjögren's syndrome OR AB Sjogrens Syndrome OR AB Syndrome, Sjogren's OR AB Sjogren Syndrome OR AB Sicca Syndrome OR AB Syndrome, Sicca

S2 AB Exercise OR AB Exercise, Physical OR AB Exercises, Physical OR AB Physical Exercise OR AB Physical Activity OR AB Exercise, Aerobic OR AB Exercise, Isometric OR AB Acute Exercise OR AB Exercise Training

S3 AB Resistance Training OR AB Strength Training OR AB Weight-Lifting Strengthening Program OR AB Strengthening Programs, Weight-Lifting OR AB Strengthening Program, Weight-Lifting OR AB Weight-Lifting Exercise Program OR AB Weight Bearing Strengthening Program OR AB Weight-Bearing Exercise Program OR AB Weight-Bearing Exercise Programs

S4 AB tai ji quan OR AB Tai Ji OR AB Tai Chi OR AB Tai Chi Chuan OR AB T'ai Chi OR AB Ji Quan, Tai

S5 AB Randomized Controlled Trials OR AB Clinical Trials, Randomized OR AB Trials, Randomized Clinical OR AB Controlled Clinical Trials, Randomized

S6 (((SU Randomized Controlled Trials as Topic OR SU ( randomized controlled trial OR randomized OR placebo )) AND (S7 OR S8 OR S9)) AND (S10 AND S11 AND S12)) AND (S2 OR S3 OR S4)

**1.7 ClinicalTrials.gov**

#1 Sjogren's syndrome OR Sjogren Syndrome OR Sjogren's Disease OR Sjogren-Larsson Syndrome OR Sjogrens Disease

#2 Exercise OR Exercise Training OR Exercise Therapy OR Exercise Performanc OR Exercise Capacity OR Exercise Addiction

#3 Physical Activity OR Physical Activity Level OR Physical Activity Behavor OR Physical Activity Levels OR Physical Activity Awareness OR Physical Activity Enjoyment

#4 Resistance Training OR Resistance Exercise OR Resistance Bacterial OR Resistance, Disease

#5 Randomized Controlled Trial OR Randomized Clinical Trial OR Randomized Controlled Trials OR Randomized Controlled Study OR Randomized Study

#6 (Sjogren's syndrome OR Sjogren Syndrome OR Sjogren's Disease OR Sjogren-Larsson Syndrome OR Sjogrens Disease) AND (Exercise OR Exercise Training OR Exercise Therapy OR Exercise Performanc OR Exercise Capacity OR Exercise Addiction AND Physical Activity OR Physical Activity Level OR Physical Activity Behavior OR Physical Activity Levels OR Physical Activity Awareness OR Physical Activity Enjoyment) AND (Resistance Training OR Resistance Exercise OR Resistance Bacterial OR Resistance, Disease) AND (Randomized Controlled Trial OR Randomized Clinical Trial OR Randomized Controlled Trials OR Randomized Controlled Study OR Randomized Study)

**Appendix 2. Risk of bias**

**2.1 Risk of bias graph**

**Figure. S1** Risk of bias graph

**2.2 Risk of bias summary**

**
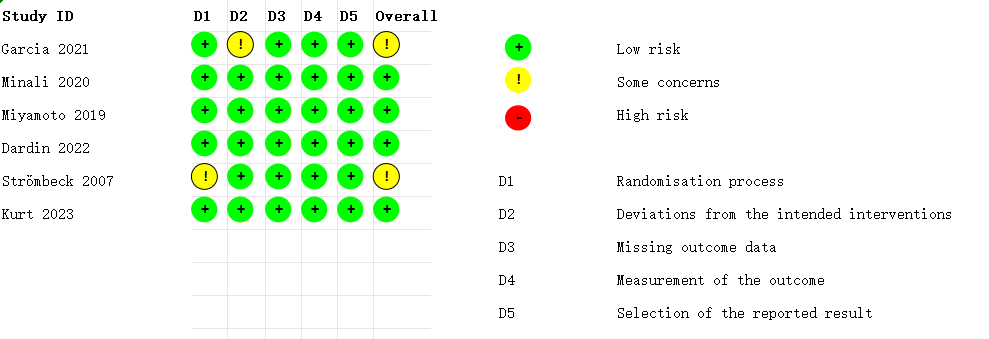
**

**Figure. S2** The detail of risk of bias

**Appendix 3. GRADE Summary of Evidence**

| Table 5 GRADE Summary of Evidence | | | | | |  |  |  |  |
| --- | --- | --- | --- | --- | --- | --- | --- | --- | --- |
|  |  |  |  |  |  | Number of Participants | |  | Quality |
| Studies | Design | Risk of  bias | Inconsistency | Indirectness | Imprecision | EG | CG | Absolute  (95% CI) |  |
| Cardiopulmonary function 5 | RCT | not serious | serious^1^ | not serious | not serious^3^ | 115 | 114 | 0.59 (0.20, 0.99 ) | ⊕⊕OO LOW |
| Functional capacity 3 | RCT | not serious | not serious | not serious | not serious^3^ | 65 | 61 | 0.69 (0.33, 1.05 ) | ⊕⊕⊕O MODERATE |
| Pain 5 | RCT | serious^2^ | serious^1^ | not serious | not serious^3^ | 111 | 106 | 0.32 (-0.09, 0.72) | ⊕OOO VERY LOW |
| General health status 4 | RCT | serious^2^ | not serious | not serious | not serious^3^ | 88 | 84 | 0.46 (0.15, 0.76) | ⊕⊕OO LOW |
| SF_36_vitality 3 | RCT | not serious | not serious | not serious | not serious^3^ | 65 | 61 | 0.51 (0.15, 0.86) | ⊕⊕⊕O MODERATE |
| SF_36_social aspects 3 | RCT | not serious | not serious | not serious | not serious^3^ | 65 | 61 | 0.27 (-0.08, 0.62) | ⊕⊕⊕O MODERATE |
| ESSDAI 3 | RCT | not serious | not serious | not serious | not serious^3^ | 65 | 61 | -0.09 (-0.41, 0.23) | ⊕⊕⊕O MODERATE |
| Fatigue 4 | RCT | not serious | serious^1^ | not serious | not serious^3^ | 71 | 68 | -0.57 (-1.57, 0.44) | ⊕⊕OO LOW |
| Mental health 8 | RCT | serious^2^ | not serious | not serious | not serious^3^ | 162 | 154 | 0.42 (0.13, 0.72) | ⊕⊕OO LOW |
| *Notes:* EG, exercise group; CG, control group; RCT, randomized controlled trial; CI, confidence interval; SF_36_vitality, 36 item short form health survey vitality; SF_36_social aspects, 36 item short form health survey social aspects; ESSDAI, EULAR Sjögren's syndrome disease activity index.  ^1^ The heterogeneity is large due to among study differences, ^2^ Number of participants drop out, ^3^ Total population size is less than 200. | | | | | | | | | |

**Appendix 4. Sensitivity Analysis and Publication Bias**

**4.1 Sensitivity Analysis**

**
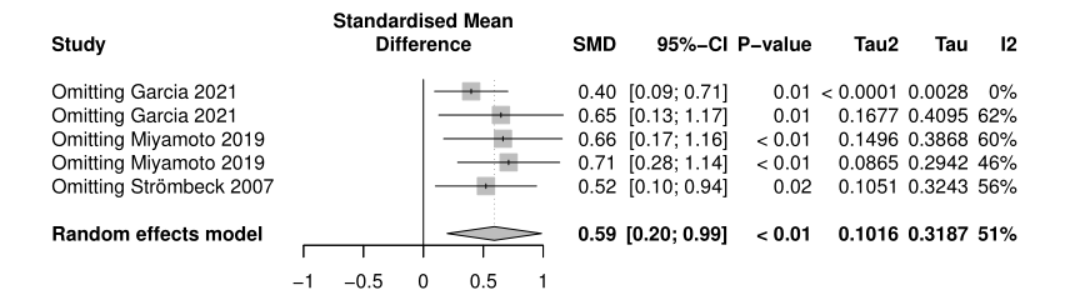
**

**Figure. S3** Based on sensitivity analysis for cardiopulmonary function. CI, confidence interval; SMD, standardised mean difference.

**
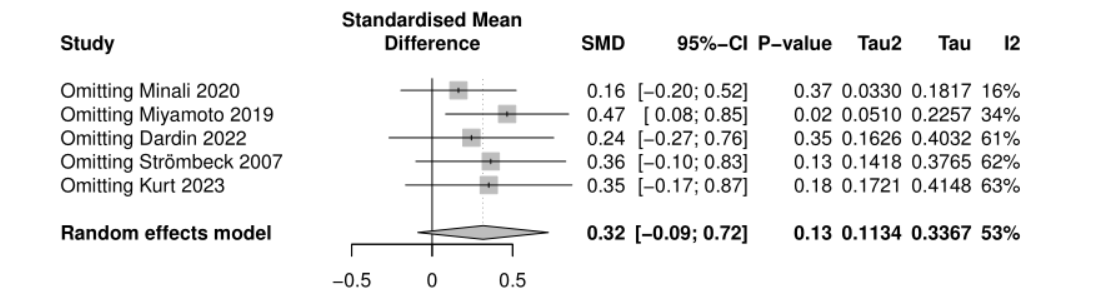
**

**Figure. S4** Based on sensitivity analysis for pain. CI, confidence interval; SMD, standardised mean difference.

**4.2 Publication Bias**

**
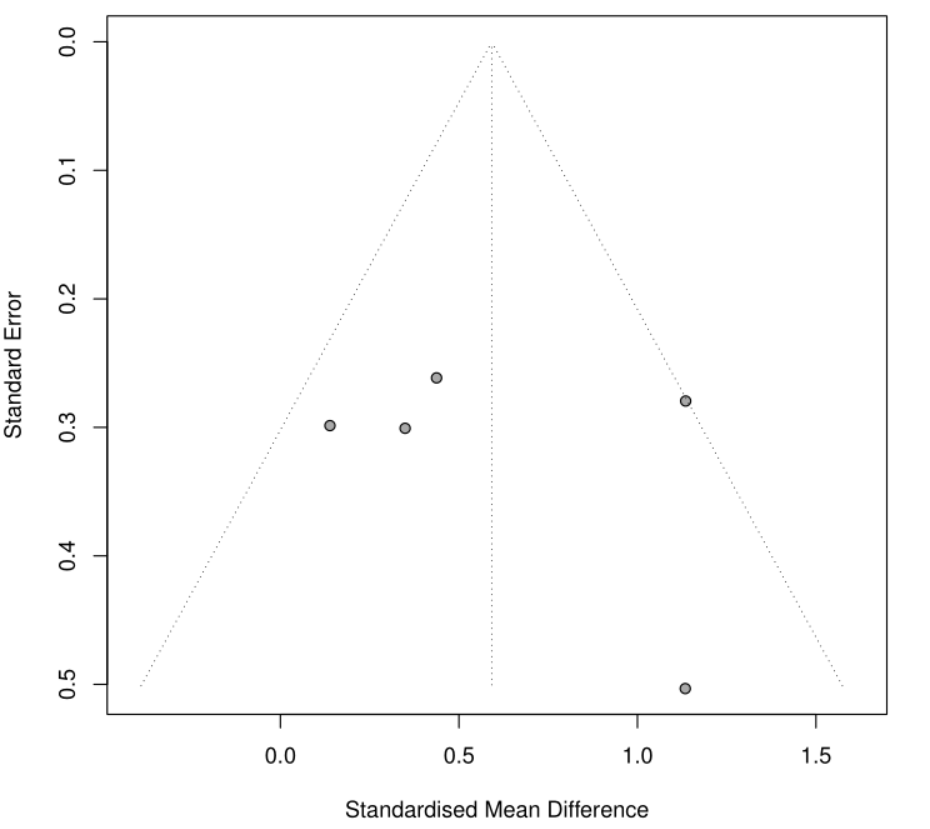
**

**Figure. S5** Funnel plot for cardiopulmonary function. SMD, standardised mean difference.

**
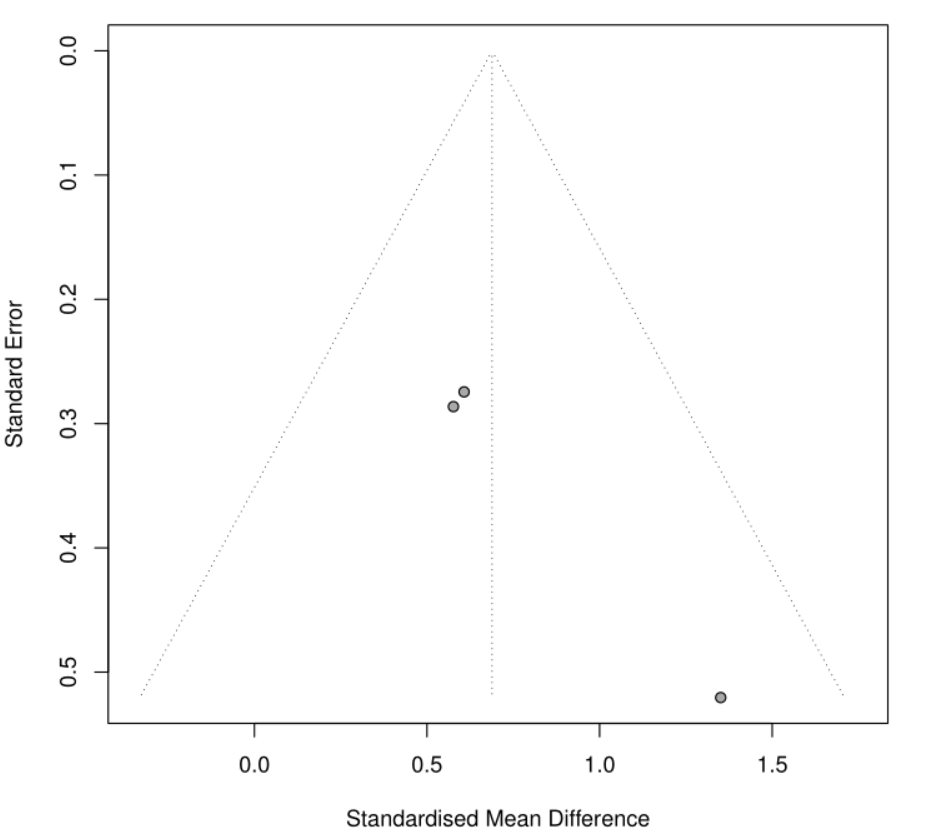
**

**Figure. S6** Funnel plot for functional capacity. SMD, standardised mean difference.

**
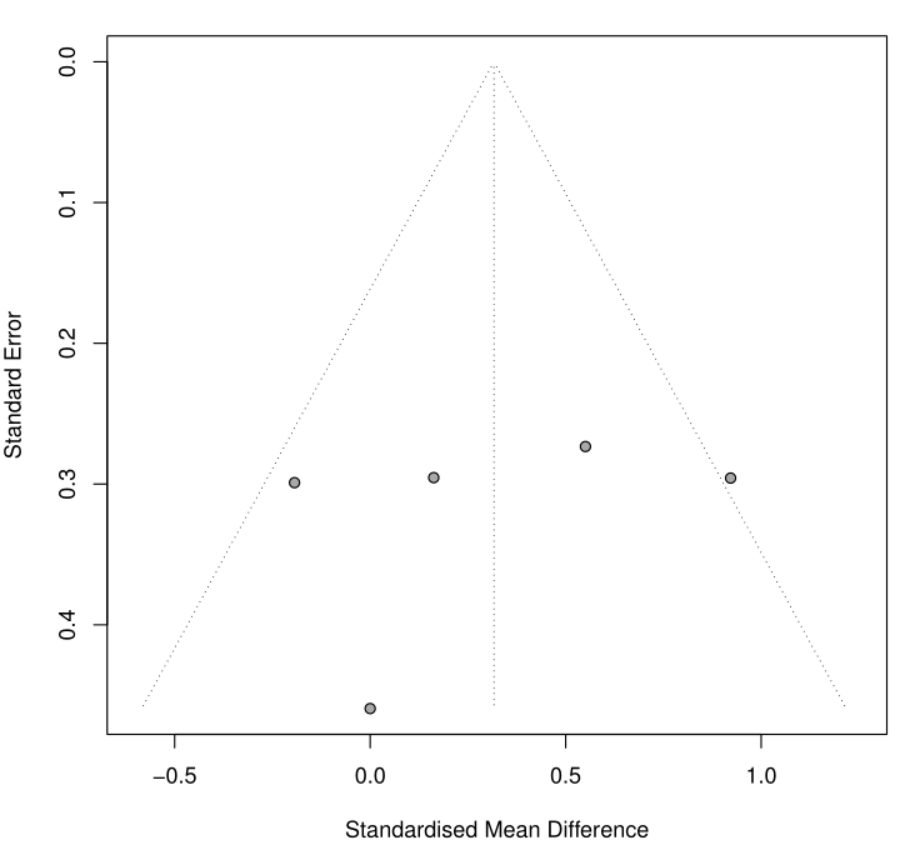
**

**Figure. S7** Funnel plot for pain. SMD, standardised mean difference.

**
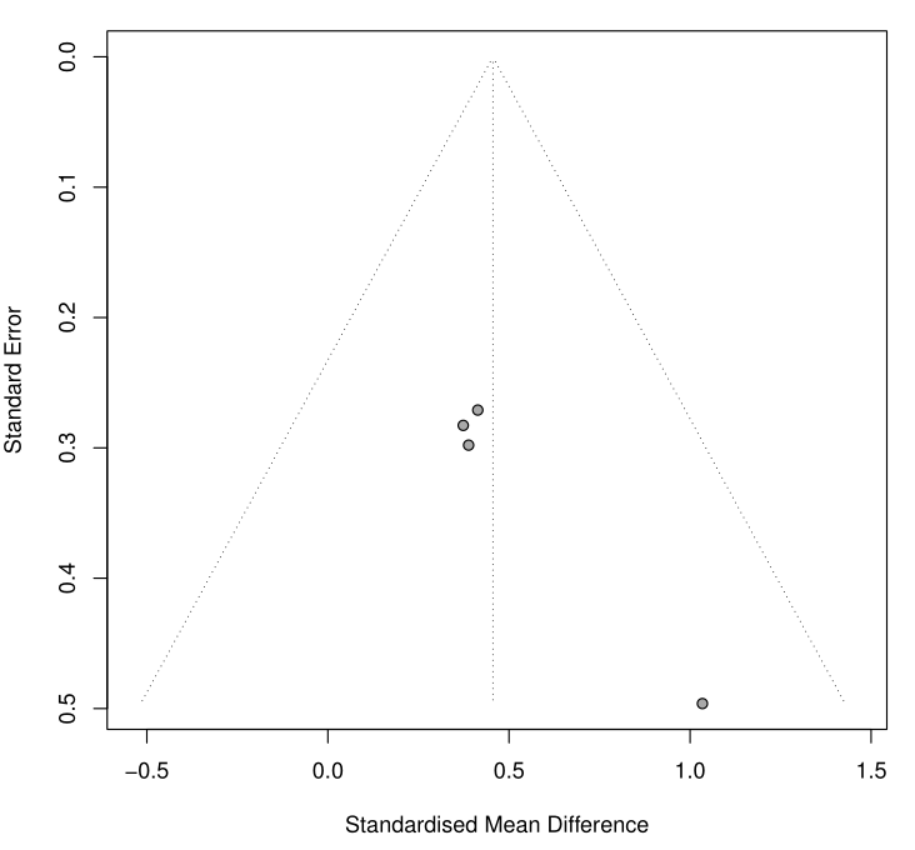
**

**Figure. S8** Funnel plot for general health status. SMD, standardised mean difference.

**
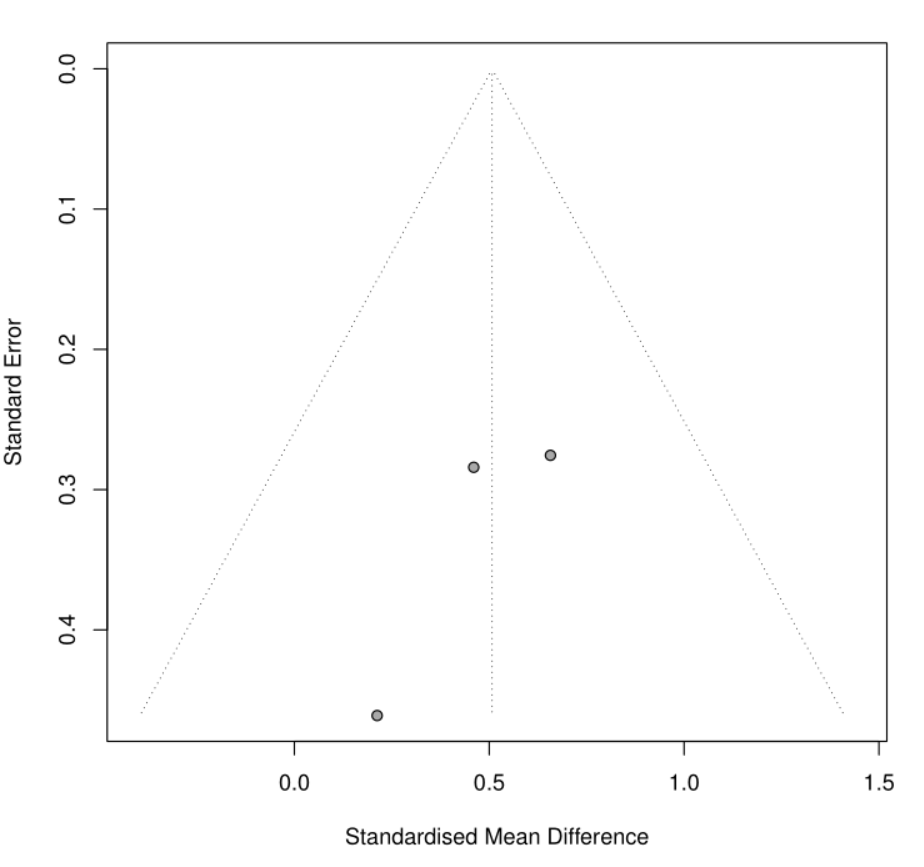
**

**Figure. S9** Funnel plot for vitality. SMD, standardised mean difference.

**
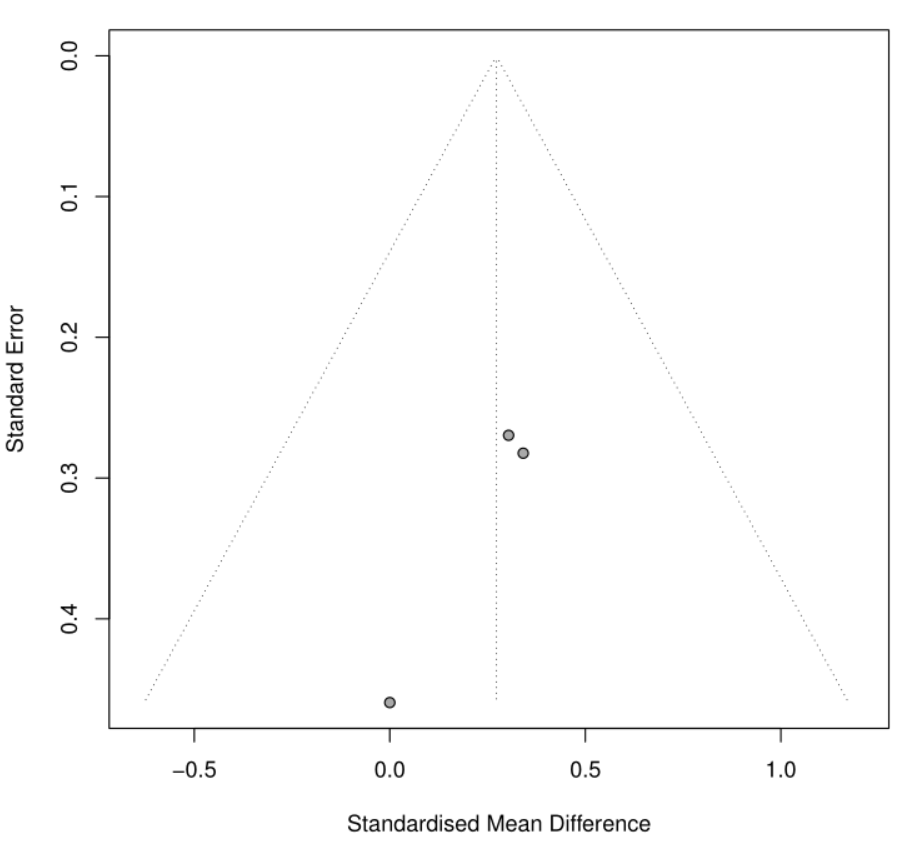
**

**Figure. S10** Funnel plot for social aspects. SMD, standardised mean difference.

**
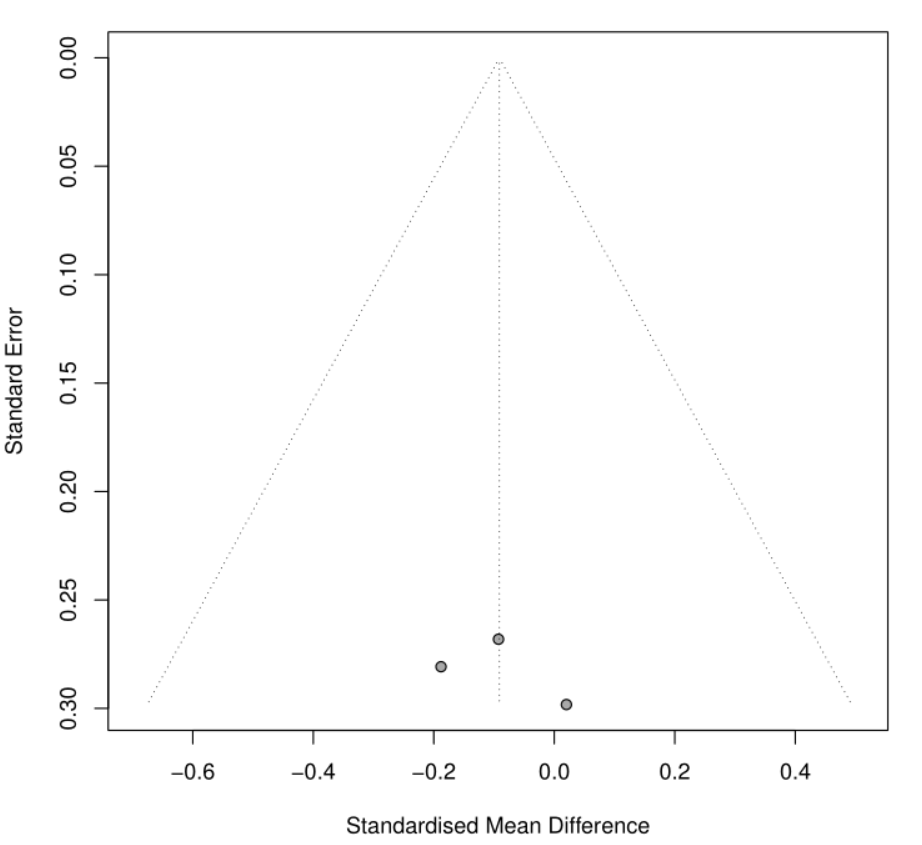
**

**Figure. S11** Funnel plot for ESSDAI. SMD, standardised mean difference.


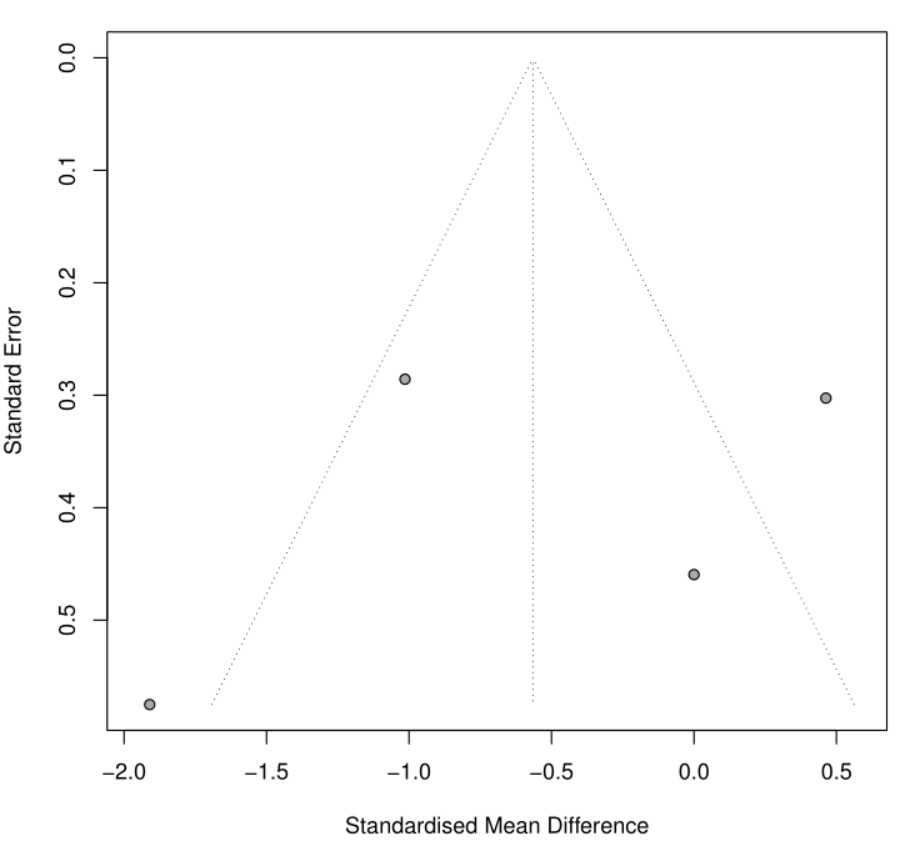


**Figure. S12** Funnel plot for Fatigue. SMD, standardised mean difference.


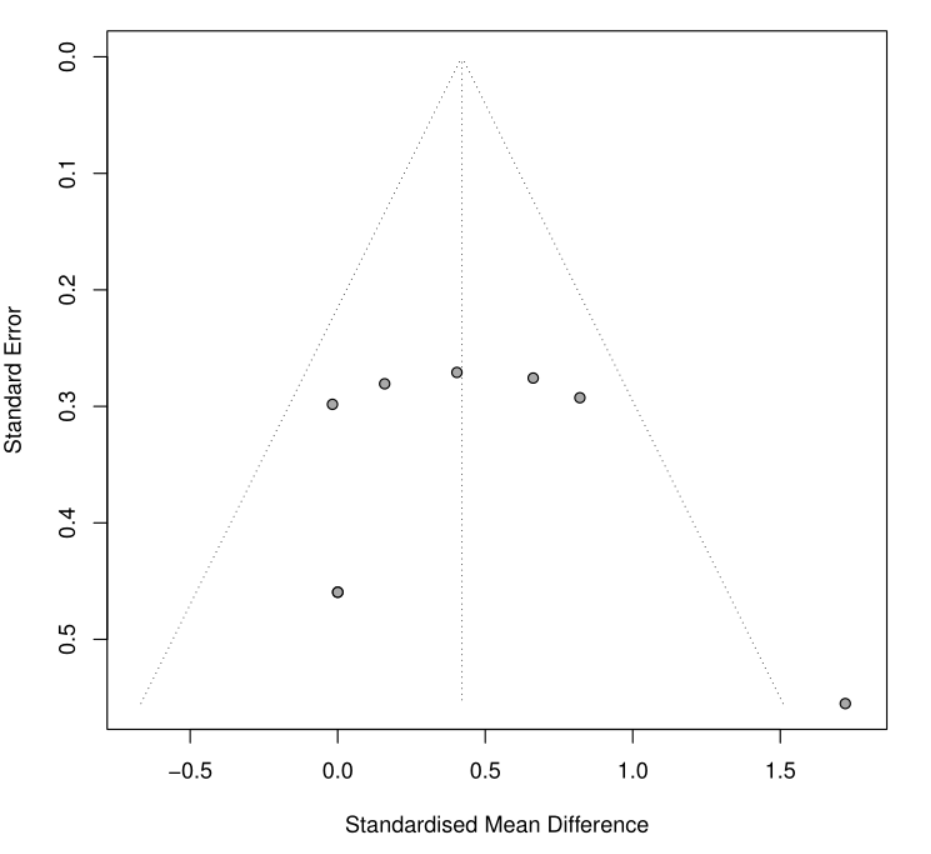


**Figure. S13** Funnel plot for Mental Health. SMD, standardised mean difference.

**Appendix 5. PRISMA 2020 Checklist**

| **Section and Topic** | **Item #** | **Checklist item** | **Location where item is reported** |
| --- | --- | --- | --- |
| **TITLE** | | |  |
| Title | 1 | Identify the report as a systematic review. | Page 1. Lines1-2 |
| **ABSTRACT** | | |  |
| Abstract | 2 | See the PRISMA 2020 for Abstracts checklist (Table 2). | Page 1-2. Lines4-23 |
| **INTRODUCTION** | | |  |
| Rationale | 3 | Describe the rationale for the review in the context of existing knowledge. | Page 3-4. Lines29-67 |
| Objectives | 4 | Provide an explicit statement of the objective(s) or question(s) the review addresses. | Page 4-5. Lines68-83 |
| **METHODS** | | |  |
| Eligibility criteria | 5 | Specify the inclusion and exclusion criteria for the review and how studies were grouped for the syntheses. | Page 6-7. Lines103-117 |
| Information sources | 6 | Specify all databases, registers, websites, organisations, reference lists and other sources searched or consulted to identify studies. Specify the date when each source was last searched or consulted. | Page 5-6. Lines87-100 |
| Search strategy | 7 | Present the full search strategies for all databases, registers and websites, including any filters and limits used. | Page 6. Lines94-100 |
| Selection process | 8 | Specify the methods used to decide whether a study met the inclusion criteria of the review, including how many reviewers screened each record and each report retrieved, whether they worked independently, and if applicable, details of automation tools used in the process. | Page 7-8. Lines120-133 |
| Data collection process | 9 | Specify the methods used to collect data from reports, including how many reviewers collected data from each report, whether they worked independently, any processes for obtaining or confirming data from study investigators, and if applicable, details of automation tools used in the process. | Page 7-8. Lines120-133 |
| Data items | 10a | List and define all outcomes for which data were sought. Specify whether all results that were compatible with each outcome domain in each study were sought (e.g. for all measures, time points, analyses), and if not, the methods used to decide which results to collect. | Page 7-10. Lines120-178 |
|  | 10b | List and define all other variables for which data were sought (e.g. participant and intervention characteristics, funding sources). Describe any assumptions made about any missing or unclear information. | Page 7-10. Lines120-178 |
| Study risk of bias assessment | 11 | Specify the methods used to assess risk of bias in the included studies, including details of the tool(s) used, how many reviewers assessed each study and whether they worked independently, and if applicable, details of automation tools used in the process. | Page 8. Lines136-142 |
| Effect measures | 12 | Specify for each outcome the effect measure(s) (e.g. risk ratio, mean difference) used in the synthesis or presentation of results. | Page 8-9. Lines145-168 |
| Synthesis methods | 13a | Describe the processes used to decide which studies were eligible for each synthesis (e.g. tabulating the study intervention characteristics and comparing against the planned groups for each synthesis (item #5)). | Page 8-10. Lines145-178 |
|  | 13b | Describe any methods required to prepare the data for presentation or synthesis, such as handling of missing summary statistics, or data conversions. | Page 8-10. Lines145-178 |
|  | 13c | Describe any methods used to tabulate or visually display results of individual studies and syntheses. | Page 8-10. Lines145-178 |
|  | 13d | Describe any methods used to synthesize results and provide a rationale for the choice(s). If meta-analysis was performed, describe the model(s), method(s) to identify the presence and extent of statistical heterogeneity, and software package(s) used. | Page 8-10. Lines145-178 |
|  | 13e | Describe any methods used to explore possible causes of heterogeneity among study results (e.g. subgroup analysis, meta-regression). | Page 8-10. Lines145-178 |
|  | 13f | Describe any sensitivity analyses conducted to assess robustness of the synthesized results. | Page 9. Lines171-173 |
| Reporting bias assessment | 14 | Describe any methods used to assess risk of bias due to missing results in a synthesis (arising from reporting biases). | Page 8. Lines136-142 |
| Certainty assessment | 15 | Describe any methods used to assess certainty (or confidence) in the body of evidence for an outcome. | Page 8. Lines136-142 |
| **RESULTS** | | |  |
| Study selection | 16a | Describe the results of the search and selection process, from the number of records identified in the search to the number of studies included in the review, ideally using a flow diagram. | Page 10. Lines182-184 |
|  | 16b | Cite studies that might appear to meet the inclusion criteria, but which were excluded, and explain why they were excluded. | Page 10. Lines182-184 |
| Study characteristics | 17 | Cite each included study and present its characteristics. | Page 10. Lines187-194 |
| Risk of bias in studies | 18 | Present assessments of risk of bias for each included study. | Page 11. Lines197-203 |
| Results of individual studies | 19 | For all outcomes, present, for each study: (a) summary statistics for each group (where appropriate) and (b) an effect estimate and its precision (e.g. confidence/credible interval), ideally using structured tables or plots. | Page 11-14. Lines208-264 |
| Results of syntheses | 20a | For each synthesis, briefly summarise the characteristics and risk of bias among contributing studies. | Page 11-13. Lines208-256 |
|  | 20b | Present results of all statistical syntheses conducted. If meta-analysis was done, present for each the summary estimate and its precision (e.g. confidence/credible interval) and measures of statistical heterogeneity. If comparing groups, describe the direction of the effect. | Page 11-13. Lines208-256 |
|  | 20c | Present results of all investigations of possible causes of heterogeneity among study results. | Page 11-13. Lines208-256 |
|  | 20d | Present results of all sensitivity analyses conducted to assess the robustness of the synthesized results. | Page 14. Lines259-264 |
| Reporting biases | 21 | Present assessments of risk of bias due to missing results (arising from reporting biases) for each synthesis assessed. | Page 8. Lines136-142 |
| Certainty of evidence | 22 | Present assessments of certainty (or confidence) in the body of evidence for each outcome assessed. | Page 8. Lines136-142 |
| **DISCUSSION** | | |  |
| Discussion | 23a | Provide a general interpretation of the results in the context of other evidence. | Page 14-17. Lines267-336 |
|  | 23b | Discuss any limitations of the evidence included in the review. | Page 18. Lines349-360 |
|  | 23c | Discuss any limitations of the review processes used. | Page 18. Lines349-360 |
|  | 23d | Discuss implications of the results for practice, policy, and future research. | Page 19  Lines363-372 |
| **OTHER INFORMATION** | | |  |
| Registration and protocol | 24a | Provide registration information for the review, including register name and registration number, or state that the review was not registered. | Page 5. Lines87-90 |
|  | 24b | Indicate where the review protocol can be accessed, or state that a protocol was not prepared. | NA |
|  | 24c | Describe and explain any amendments to information provided at registration or in the protocol. | NA |
| Support | 25 | Describe sources of financial or non-financial support for the review, and the role of the funders or sponsors in the review. | NA |
| Competing interests | 26 | Declare any competing interests of review authors. | NA |
| Availability of data, code and other materials | 27 | Report which of the following are publicly available and where they can be found: template data collection forms; data extracted from included studies; data used for all analyses; analytic code; any other materials used in the review. | NA |
